# A Network‐Based Association of IBD and Colorectal Cancer Using Proteomics Data

**DOI:** 10.1002/prca.70041

**Published:** 2026-02-17

**Authors:** Jaiya Dhami, Swarnima Kollampallath Radhakrishnan, Dominic Russ, Sudip Mondal, Abdulrahman Alzarooni, Laura Bravo Merodio, Niharika A. Duggal, Ruchi Gupta, Animesh Acharjee

**Affiliations:** ^1^ Cancer and Genomic Sciences School of Medical Sciences College of Medicine and Health University of Birmingham UK; ^2^ Centre For Health Data Research University of Birmingham Birmingham UK; ^3^ Institute of Translational Medicine University Hospitals Birmingham NHS Foundation Trust Birmingham UK; ^4^ MRC‐Versus Arthritis Centre For Musculoskeletal Ageing Research Birmingham UK; ^5^ Institute of Inflammation and Ageing University of Birmingham Birmingham UK; ^6^ School of Chemistry University of Birmingham Birmingham UK

**Keywords:** IBD, colon cancer, network analysis, UK‐Biobank, omics

## Abstract

**Background:**

Colorectal cancer (CRC) is a major cause of morbidity and mortality, with chronic inflammation from inflammatory bowel disease (IBD) representing a well‐established risk factor. Clarifying shared molecular mechanisms may facilitate early detection and prevention strategies.

**Methods:**

Proteomic data from the UK Biobank were analysed using the Olink proximity extension assay for seven CRC‐associated proteins (TFF3, TFF1, AHCY, RETN, LCN2, SELE and CEACAM5) previously identified via machine learning. Expression levels in CRC and IBD cases were compared with controls. Multilayer interaction networks, incorporating protein–protein, protein–metabolite and transcription factor–protein interactions, were generated using OmicsNet. Findings were validated in the Colonomics transcriptomic dataset.

**Results:**

All seven proteins were significantly upregulated in CRC; six (excluding CEACAM5) were also elevated in IBD. Network analysis identified AHCY and LCN2 as central hubs linking inflammatory and metabolic pathways. NF‐κB and GATA2 emerged as recurrent transcriptional regulators. Colonomics validation confirmed upregulation of AHCY, LCN2 and SELE in CRC tissues.

**Conclusions:**

This multi‐omics network analysis reveals a shared molecular framework between IBD and CRC, with inflammation as a key driver of colorectal carcinogenesis.

## 1 Introduction

Colorectal cancer (CRC) constitutes a significant global health challenge, ranking third in worldwide cancer incidence with 1.9 million new cases and over 900,000 deaths reported in 2022[1]. Incidence is higher in developed countries due to dietary habits and sedentary lifestyles [1, 2]. It is projected that CRC incidence is expected to reach 3 million by 2040, with developing nations experiencing a substantial increase linked to western lifestyle adoption and a rise in life expectancy [3].

**FIGURE 1:**
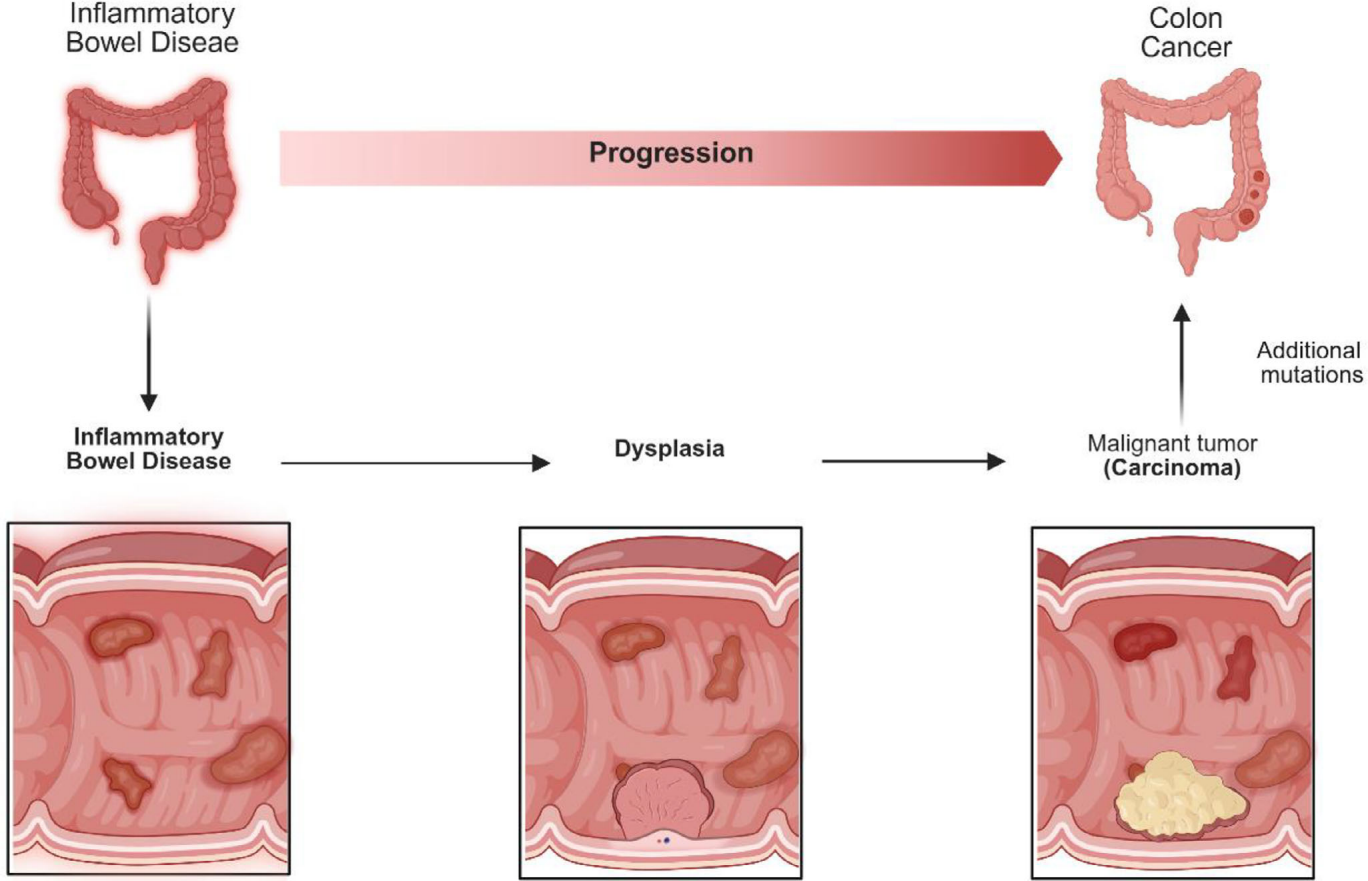
Pathway of IBD into CRC. The inflammation, dysplasia and carcinoma sequence highlights the progression from IBD to CRC through chronic inflammation and additional mutations. Produced in https://BioRender.com.

CRC arises through the gradual accumulation of genetic and epigenetic alterations, often from precancerous polyps such as adenomas and serrated polyps, which have high malignant potential [3]. The classical adenoma–carcinoma sequence involves APC, KRAS and TP53 mutations, while the serrated pathway is characterised by BRAF mutations and CpG island methylator phenotype (CIMP) progresses more rapidly following dysplasia [4, 5]. Another development pathway for tumorigenesis is chronic intestinal inflammation, particularly in the context of inflammatory bowel disease (IBD), where the inflammation‐dysplasia‐carcinoma sequence results from early TP53 mutations and chromosomal instability (Figure 1) [6, 7].

Non‐modifiable CRC risk factors include age, gender and genetic syndromes such as lynch syndrome and familial adenomatous polyposis (FAP) [8]. Lynch syndrome involves germline mutations in mismatch repair genes, leading to microsatellite instability and a 30%–70% lifetime CRC risk [9]. FAP results from an inherited APC gene mutation, with nearly 100% lifetime risk of CRC if untreated [9]. Modifiable risk factors include smoking, alcohol, obesity, type 2 diabetes and diets high in red and processed meats [10, 11, 12, 13, 14, 15]. Oppositely, high fibre intake, physical activity and medications, like aspirin, display protective properties against CRC [16, 17, 18].

Early detection through screening significantly reduces CRC mortality, with the faecal immunochemical test (FIT) now being the standard in the UK for individuals aged 54 to 74 [19, 20]. FIT has enhanced sensitivity compared to previous methods, resulting in better detection rates due to increased public uptake [21]. However, due to the nature of FIT being unpleasant the development of blood‐based markers and testing may increase early detection. Despite these advances, early‐onset CRC is rising in younger populations, often diagnosed at later stages due to symptom crossover with other gastrointestinal diseases [22, 23].

IBD, including Crohn's disease and ulcerative colitis, is a chronic, incurable inflammatory disorder of the gastrointestinal tract and an established risk factor for CRC [24, 25]. As of 2019, IBD affects 4.9 million individuals worldwide, demonstrating a 47% increase in prevalence since 1990 [26]. Patients with extensive colitis or early IBD onset tend to face a significantly elevated CRC risk, up to 70% higher than the general population [24, 27]. Chronic inflammation from IBD drives epithelial damage, increasing mutation rates, which promotes carcinogenesis. Diagnostic delays are common for CRC as the symptoms like rectal bleeding and abdominal pain overlap with IBD flare‐ups.

The intricate relationship between constant inflammation and the development of CRC is a pivotal area of research. While traditional molecular studies have found many potential biomarkers, they often do not fully represent the multifaceted nature of tumour biology. Multi‐omics integrates various high‐throughput omics technologies like genomics, transcriptomics, proteomics and metabolomics to provide a systems‐level overview of disease [28]. Multi‐omics techniques enhance cancer research by refining tumour classification, improving the understanding of disease mechanisms, facilitating biomarker discovery and optimising personal medicine [29, 30].

Proteomics focuses on quantifying protein expression and interactions, crucial in CRC due to its heterogeneity [31, 32]. Transcriptomics analyses RNA expression and has contributed to defining consensus molecular subtypes (CMS1‐4) that differ in prognosis and treatment response [33]. Individual proteins do not function in isolation; they are integrated into complex networks involving other proteins, transcription factors and metabolites [34]. Understanding these networks is essential for revealing the mechanistic bridge between IBD‐driven inflammation and CRC progression.

This study focuses on seven CRC‐associated proteins identified in the UK Biobank (TFF3, TFF1, AHCY, RETN, LCN2, SELE and CEACAM5) through machine learning‐based biomarker selection in a previous study by Radhakrishna et al. [35]. We hypothesise that these proteins are functionally interconnected within molecular networks and that elements of these networks also operate in IBD, suggesting shared mechanisms driving inflammation induced tumorigenesis.

To explore this a multi‐omics approach was applied (1) the expression of the seven proteins in CRC and IBD using proteomic data from the UK Biobank, (2) constructed molecular interaction networks covering protein–protein, metabolite–protein and transcription factor–protein relationships using OmicsNet, (3) validated findings in independent datasets and (4) investigated how these networks may reflect inflammatory mechanisms that contribute to CRC development.

### 1.1 Datasets and Methods Used

**FIGURE 2:**
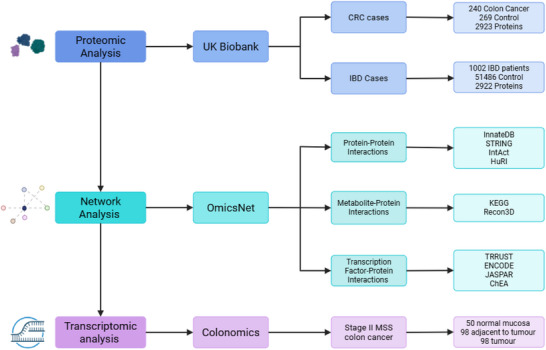
Overview of the multi‐omics study design. Proteomic data from CRC and IBD cohorts in the UK Biobank were analysed to identify differentially expressed proteins. Molecular interaction networks were constructed using OmicsNet across protein–protein, metabolite–protein and transcription factor–protein layers with the figure highlighting the databases used. Transcriptomic validation was performed using the Colonomics dataset of stage II MSS colon cancer samples. Produced in https://BioRender.com.

The design of this multi‐omics study and analysis workflow is summarised in Figure 2. The study utilises proteomic data from the UK Biobank to identify protein expression patterns in CRC and IBD cases.

### 1.2 Proteomic Data From the UK Biobank

Data were obtained from the UK Biobank, a prospective population‐based cohort study comprising 500,000 individuals [36]. Participants aged 40–69 were recruited between 2006 and 2010 across 22 assessment centres in the United Kingdom, where they were selected based on geographic proximity. The cohort was followed longitudinally for a wide range of health outcomes, including cancer, cardiovascular disease and inflammatory conditions, producing a vast database of phenotypic and omics data that provides an ideal source for proteomics studies.

### 1.3 Case and Control Data Matching

CRC and IBD case and control cohorts were derived from UK Biobank using ICD‐10 diagnostic codes. A total of 9890 participants were identified to have CRC. This was further filtered with 6372 participants having CRC from multiple sources and 3518 from a single source. Of the 3518 CRC participants with a single source, the ICD‐10 were registered from different sources: 2737 hospital records, 382 self‐reported, 325 cancer registries and 74 death registries. Age and sex matched controls without ICD‐10 diagnosis for CRC diagnosis were selected using the MatchIt package in R. This resulted in a dataset of 19,784 participants (9890 case and 9894 controls). This was further filtered to data from 509 individuals with complete metabolite (as of July 2021) and proteomic (as of July 2023), as performed in Radhakrishnan et al. [35].

For IBD, 1002 cases matched the inclusion criteria based on relevant ICD‐10 codes for IBD. These were compared to 51,486 healthy control individuals without a diagnosis of IBD. Proteomic data for CRC and IBD participants and controls were from plasma samples which were analysed using the Olink high‐throughput platform. Controls were healthy individuals with no diagnosis of CRC or IBD, selected from the UK Biobank. The data selection steps are shown in the Figure S10.

### 1.4 Rationale for Protein Selection

Seven proteins (TFF3, TFF1, AHCY, RETN, LCN2, SELE and CEACAM5) were selected for further analysis based on findings from a recent machine learning‐based study from Radhakrishnan et al. [35]. This study utilised machine learning LASSO, XGBoost and LightGBM classifiers with SHAP analysis to identify proteins that are the most predictive of CRC. These seven proteins continuously emerged as key biomarkers across multiple models.

### 1.5 Construction of Molecular Interactions

To investigate the molecular interactions associated with the seven target proteins (TFF3, TFF1, SELE, AHCY, RETN, LCN2 and CEACAM5), an in silico network‐based analysis was performed using OmicsNet 2.0 (https://www.omicsnet.ca/). OmicsNet 2.0 is a web‐based platform that enables multi‐omics data integration and interactive network visualisation [37]. Protein–protein, protein–metabolite and transcription factor–protein interactions were mapped to characterise the molecular associations of the seven selected proteins.

The seven proteins were input as seed nodes to construct interaction networks. Protein–protein interactions were obtained from InnateDB [38], STRING [39], IntAct [40] and HuRI [41]. Metabolite protein interactions were from KEGG [42] and Recon3D [43]. Transcription factor–protein interactions were from TTRUST [44], ENCODE [45], JASPAR [46] and ChEA [47].

### 1.6 Network Topology Analysis

A network topology analysis was performed to explore the structural organisation of the interaction networks. Each network outputs the number of nodes and edges representing the interactions and seeds, which are the inputted proteins. Two centrality measures were applied: degree and betweenness [48].

Degree centrality measures the number of direct connections a node has within the network, representing its local importance. Nodes with high degree values are considered to be hubs and often participate in biological functions due to their extensive direct interactions.

Betweenness centrality is a global measure that quantifies how frequently a node is on the shortest path between other nodes. Unlike degree, it depicts a node's role in connecting network regions. Nodes with high betweenness may not have many direct links but often act as bridges or bottlenecks, facilitating the flow across functional modules [48].

In this analysis, proteins exhibiting a high degree and betweenness were prioritised for further investigation, as their topological prominence suggests potential roles in molecular regulation, pathway integration or disease progression.

### 1.7 Transcriptomic Validation via Colonomics

Colonomics (https://www.colonomics.org/) is a multi‐omics database combining genotyping, DNA methylation, gene expression, miRNA expression, whole exome sequencing and copy number variations [49]. The dataset contains 100 paired tumour and adjacent normal tissues, which were collected 10 cm from the tumour margin, from patients diagnosed with stage II microsatellite stable (MSS) colon cancer. Additionally, 50 healthy colon mucosa samples were obtained from individuals who underwent colonoscopies and detected no lesions.

Gene expression profiling was performed using Affymetrix microarrays. In this study, gene expression levels were investigated for each of the seven CRC associated proteins. Gene expression levels were compared across normal mucosa, adjacent tissue and tumour tissue to assess differential expression. Further analyses were conducted on gender, CMS, BRAF V600E mutation, KRAS mutation and CIMP status.

These findings were subsequently used to validate the findings of the UK Biobank and deepen the understanding and biological relevance of the results.

### 1.8 Statistical Analysis

For the UK Biobank colon cancer dataset, descriptive statistics (mean ± standard error of the mean (SEM)) were calculated for the expression of seven proteins in case and control groups. For the IBD dataset, descriptive statistics (mean ± SEM) were calculated for six proteins. CEACAM5 was not found and considered missing. Protein expression values were normalised through autoscaling, where each value was transformed by subtracting the variable's mean and dividing it by its standard deviation (SD). This ensures a comparative analysis and reduces potential bias. Due to the large datasets, normality was assumed based on the Central Limit Theorem [50]. Therefore, a comparison between the case and control was conducted using a one‐tailed Student's *t*‐test to assess upregulation in protein expression for the case compared to the control. Proteomic statistical analyses were performed using R (version 4.4.2), and graphical visualisations were created using GraphPad Prism (version 10.4.2).

For the Colonomics dataset, descriptive statistics (mean ± SD) were derived for gene expression across tissue types, molecular subtypes, mutation status and clinical features. We have used two‐tailed testing and FDR correction using the Benjamini–Hochberg (BH) method. Statistical significance was set at *p* < 0.05 (FDR corrected).

## 2 Results

### 2.1 Protein Expression in Colon Cancer

**FIGURE 3:**
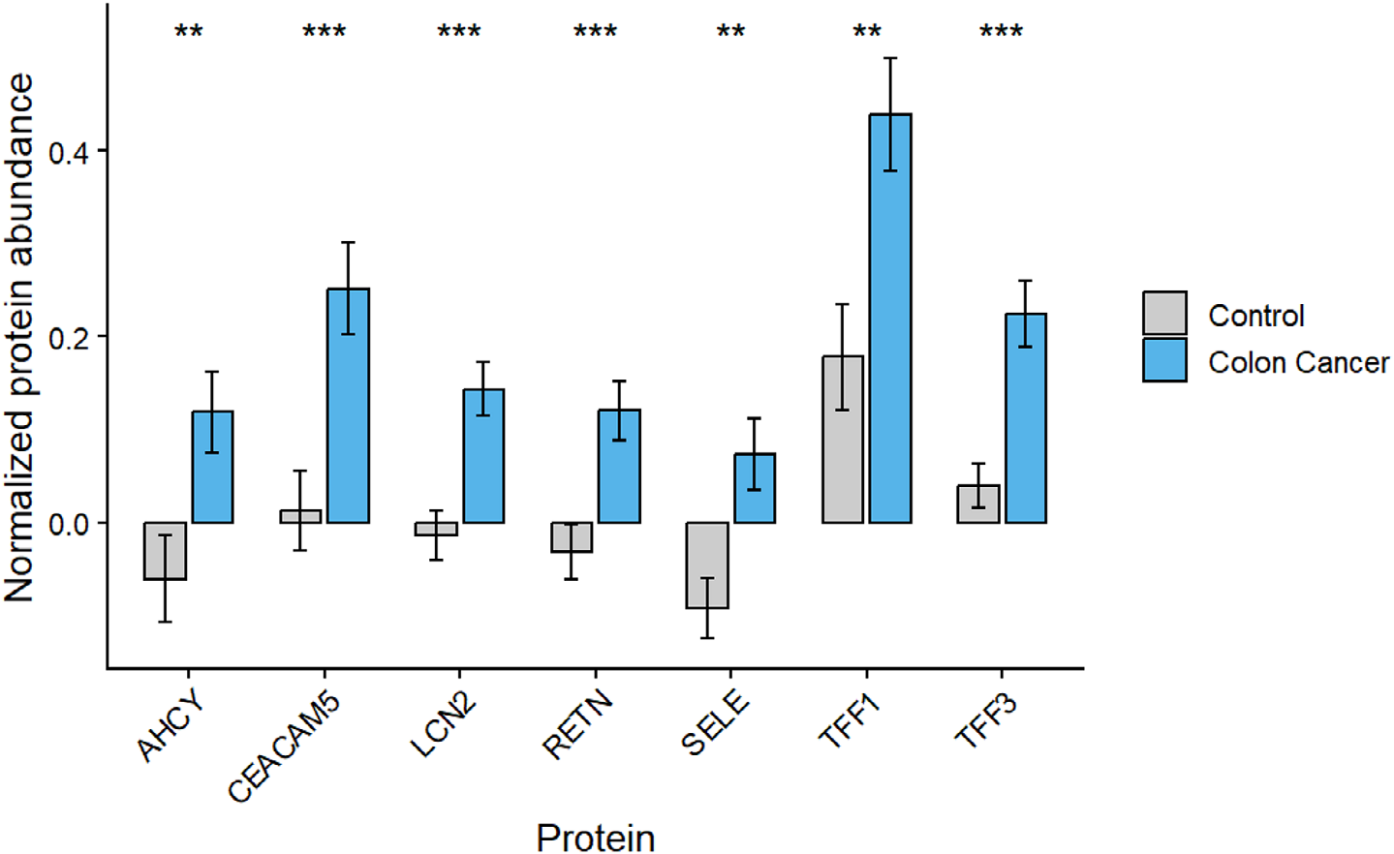
Mean protein expression in colon cancer and control samples. Protein expression of seven CRC associated proteins (TFF3, TFF1, SELE, RETN, LCN2, AHCY and CEACAM5) in colon cancer (*n* = 269) and control (*n* = 240) groups from UK Biobank. Expression values have been normalised using autoscaling (mean centred and divided by SD). Bars represent mean with error bars representing ± SEM. Grey bars represent the control, blue bars represent CRC samples. * *p* < 0.05.

Protein expression levels of seven CRC associated proteins (TFF3, TFF1, SELE, RETN, LCN2, AHCY and CEACAM5) were analysed in control and colon cancer samples from the UK Biobank. All seven proteins demonstrated significantly elevated expression in colon cancer cases compared to the control (*p* < 0.05) (Figure 3). TFF1 displayed the highest mean expression in both groups (colon cancer = 0.43 and control = 0.17), whereas SELE had the lowest average expression across both groups (colon cancer = 0.07 and control = −0.091). SELE, RETN, LCN2 and AHCY showed negative protein expression levels in control samples due to the normalisation of the dataset. Nevertheless, all CRC samples exhibited positive mean expression for all proteins.

### 2.2 Protein Expression in IBD

**FIGURE 4:**
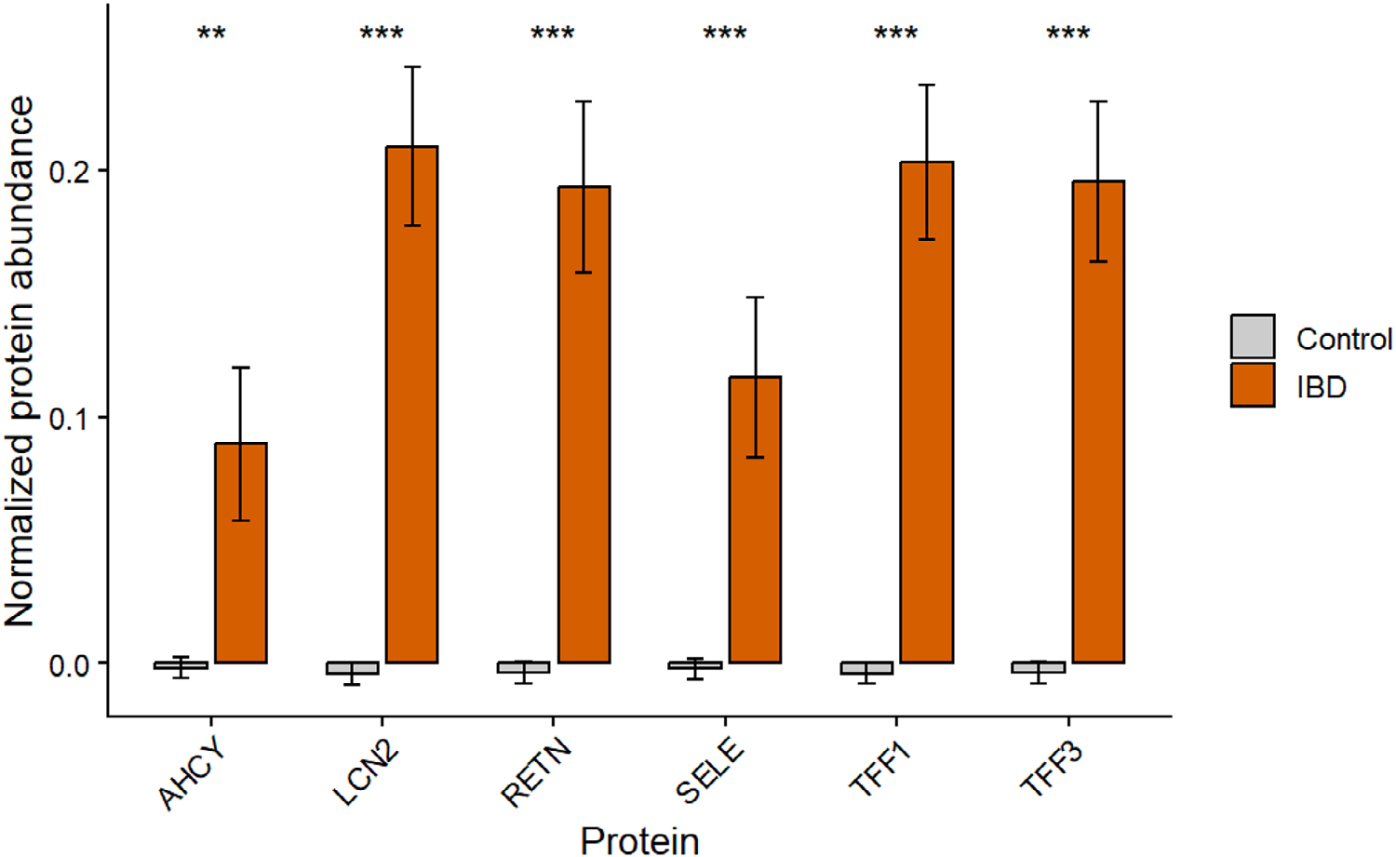
Mean protein expression in IBD and control samples. Protein expression of six CRC associated proteins (TFF3, TFF1, SELE, RETN, LCN2 and AHCY) in IBD from UK Biobank. IBD cases (*n* = 1002) are represented in green and controls (*n* = 51,486) are represented in grey bars. CEACAM5 was not present in the dataset. Expression values have been normalised using autoscaling (mean centred and divided by SD). Bars represent mean expression ± SEM. * *p* < 0.05.

To investigate the potential intersection between CRC and IBD, the expression of six CRC associated proteins (TFF3, TFF1, AHCY, RETN, LCN2 and SELE) were analysed in IBD cases (*n* = 1002) and control (*n* = 51486) groups from the UK Biobank dataset (Figure 4). CEACAM5 was not detected in this dataset for IBD. All six proteins displayed a significant increase in expression for IBD samples (*p* < 0.05) compared to control samples. All control samples had negative or near zero expression, due to normalisation. LCN2 had the highest mean expression out of the IBD cases. These results present a strong upregulation of these six CRC associated proteins in IBD.

### 2.3 Protein–Protein Interactions

To explore molecular interactions between seven CRC‐associated proteins (TFF3, TFF1, AHCY, RETN, LCN2, SELE and CEACAM5), PPI networks were constructed using OmicsNet via four databases: InnateDB, STRING, IntAct and Huri.

**FIGURE 5:**
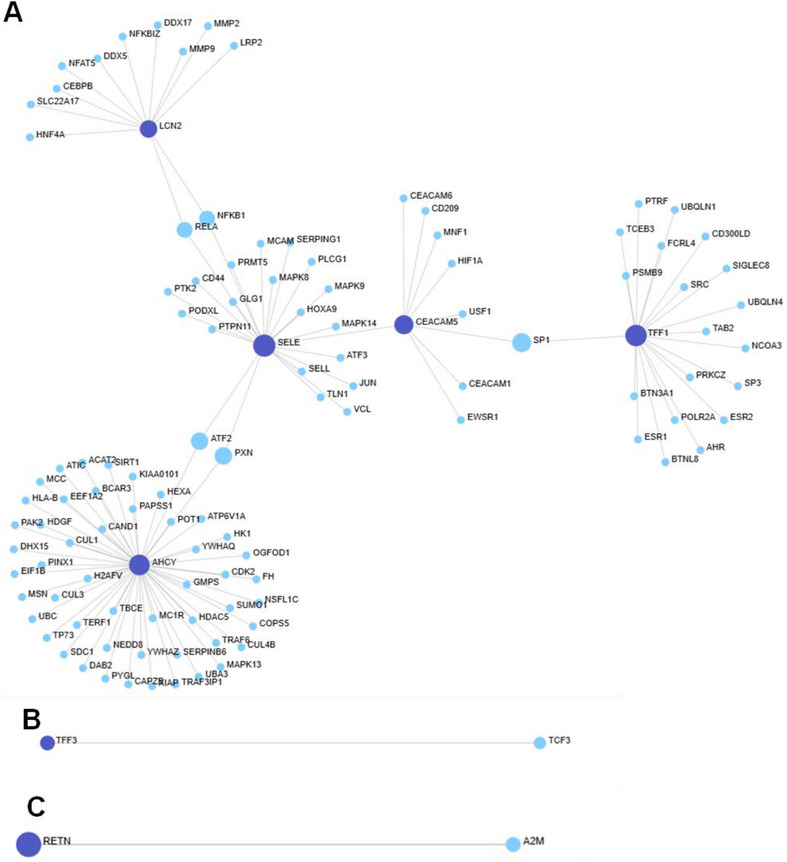
InnateDB PPI Network. PPI network generated using InnateDB from seven CRC associated (TFF3, TFF1, AHCY, RETN, SELE, LCN2 and CEACAM5) seed proteins. The network contains all seven seed proteins with 118 nodes and 117 edges across three subnetworks. (A) The larger subnetwork contains five of the seed proteins (AHCY, SELE, TFF1, LCN2 and CEACAM5 in decreasing centrality measures). AHCY presented as the central node with the highest degree (52) and betweenness (4375.5). TFF3 (B) and RETN (C) produced their own subnetworks, each containing a single seed protein with a direct interaction. Dark blue nodes indicate seed proteins, light blue nodes represent proteins. Grey edges represent direct protein–protein inteactions. Network visualised using OmicsNet.

In InnateDB (Figure 5), 118 nodes and 117 edges formed three subnetworks. AHCY emerged as the central hub (degree = 52 and betweenness = 4375.5), followed by SELE, TFF1 and CEACAM5. TFF3 and RETN showed minimal connectivity (degree = 1). Key non‐seed interactors included SP1, ATF2, RELA and NF‐κB, indicating regulatory relevance.

STRING (Supporting Information) generated the smallest and most fragmented network (31 nodes, 50 edges). Each protein formed isolated subnetworks. LCN2 was most connected (degree = 10), while CEACAM5, TFF1 and TFF3 had minimal interaction. RETN was not detected.

**FIGURE 6:**
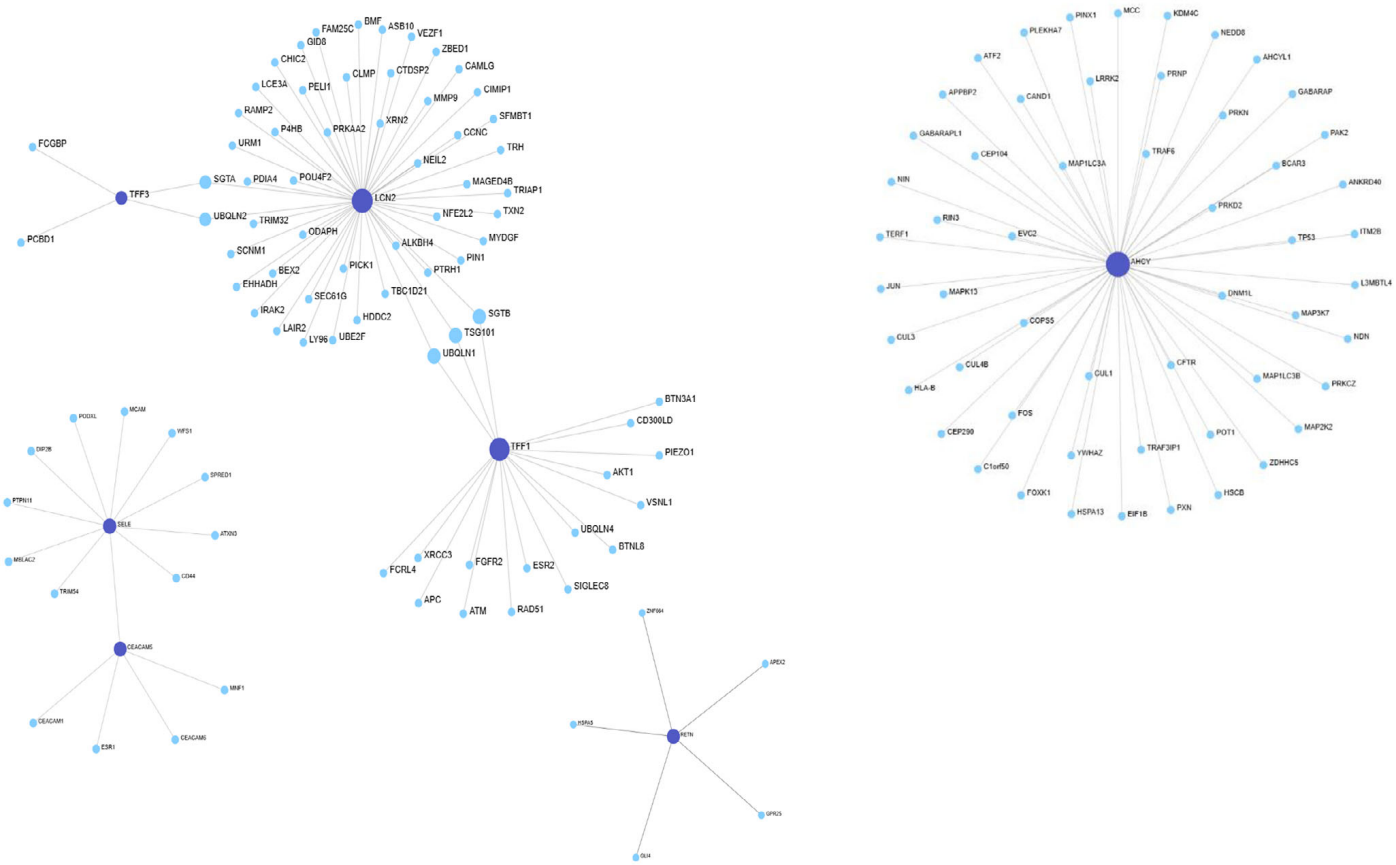
IntAct PPI Network. PPI network produced using IntAct for the seven CRC associated seed proteins (TFF3, TFF1, AHCY, RETN, SELE, LCN2 and CEACAM5). The network consisted of 148 nodes and 195 edges across four subnetworks. (A) The largest subnetwork containing 71 proteins and 73 interactions including LCN2, TFF1 and TFF3. LCN2 exhibited the highest centrality in this subnetwork (degree = 52 and betweenness = 931). (B) AHCY formed an independent subnetwork with 54 interactions (degree = 54 and betweenness = 1431) which was the highest out of all the seed proteins In IntAct. (C) RETN formed a separate minimally connected subnetwork with five integrating proteins (degree = 5 and betweenness = 10). (D) SELE and CEACAM5 existed together in a moderate subnetwork. SELE displayed moderate connectivity whereas CEACAM5 was slightly less. IntAct was the only database in which all seed proteins demonstrated nonzero betweenness values. Dark blue nodes indicate seed proteins, light blue nodes represent proteins. Grey edges represent direct protein–protein interactions. Network visualised using OmicsNet.

IntAct (Figure 6) presented the largest and most integrated network (148 nodes, 195 edges). All seven proteins were included. AHCY and LCN2 were central across separate subnetworks. TFF1 showed moderate and RETN minimal connectivity. Notably, all seed proteins had nonzero betweenness, indicating both local and global network roles. Additional hubs included SGTA, UBQLN1 and UBQLN2

HuRI (Supporting Information) formed a 66‐node network with three subnetworks. LCN2 again acted as a central hub (degree = 57). TFF1 and TFF3 were peripherally connected. RETN and AHCY each formed isolated single‐interaction subnetworks. SELE and CEACAM5 were not detected.

In summary, AHCY and LCN2 consistently emerged as key hubs, with TFF3 and RETN showing low connectivity. Network structure and protein centrality varied by database, highlighting the need for multisource validation.

### 2.4 Metabolite–Protein Interactions

**FIGURE 7:**
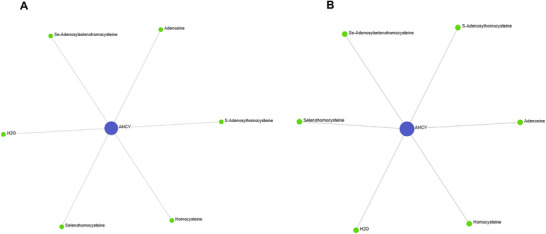
Metabolite–protein interaction networks (KEGG and Recon3D). Metabolite–protein interaction networks generated using KEGG (A) and Recon3D (B) for seven CRC associated seed proteins (TFF3, TFF1, AHCY, RETN, LCN2, SELE and CEACAM5) via OmicsNet. Only AHCY demonstrated interactions with metabolites in both datasets. Both networks portray AHCY interacting with the same six metabolites (*S*‐adenosylhomocysteine, homocysteine, adenosine, H_2_O, Se‐adenosylselenohomocysteine and selenohomocysteine). Dark blue nodes represent the seed proteins, green nodes represent metabolites. Grey edges represent direct metabolite–protein interactions. Network visualised using OmicsNet.

To investigate potential metabolite–protein interactions (MPIs), all seven CRC associated proteins (TFF3, TFF1, AHCY, RETN, LCN2, SELE and CEACAM5) were inputted into KEGG and Recon3D on OmicsNet (Figure 7). In both databases, only AHCY was found to interact with metabolites, forming a metabolic hub, while the other six proteins displayed no associations. In the KEGG MPI network (Figure 9A) AHCY interacted with six metabolites (S adenosylhomocysteine, homocysteine, adenosine, H_2_O, Se‐adenosylselenohomocysteine and selenohomocysteine). Centrality analysis revealed AHCY as a dominant hub with a degree of 6 and a betweenness centrality of 15. All six metabolites were peripheral nodes (degree = 1 and betweenness = 0), indicating AHCY as a central metabolic enzyme. The Recon3D network (Figure 9B) replicated these findings with the same six metabolites identified. AHCY also have the same degree and betweenness as well as the metabolites having the same centrality measures. This supports the robustness of AHCY metabolic interactions and reduces potential database bias. This reproducibility from independent metabolic databases reinforces the biological relevance of AHCY in CRC‐associated metabolism.

### 2.5 Transcription Factor—Protein Interactions

To investigate transcriptional regulation of seven CRC‐associated proteins (TFF3, TFF1, AHCY, RETN, LCN2, SELE and CEACAM5), transcription factor–protein interaction (TFPI) networks were generated using TTRUST, ENCODE, JASPAR and ChEA via OmicsNet.

TTRUST (Supporting Information) produced 38 nodes across three subnetworks. The largest included TFF1, SELE, TFF3 and LCN2, linked to shared transcription factors (TF) such as RELA, NF‐κB, SP1 and STAT1. TFF1 was the most connected (degree = 17 and betweenness = 330.25), followed by SELE and TFF3. RETN and CEACAM5 formed smaller, minimally connected subnetworks, while AHCY was not detected.

ENCODE (Supporting Information) revealed 122 nodes and 134 edges, with six proteins in the main network (AHCY, LCN2, TFF1, TFF3, SELE and CEACAM5). AHCY was the most transcriptionally regulated (degree = 67 and betweenness = 5241.7), followed by LCN2 and TFF1. RETN formed an isolated subnetwork with only two TFs (REST, LSMBTL2), indicating low regulatory integration.

**FIGURE 8:**
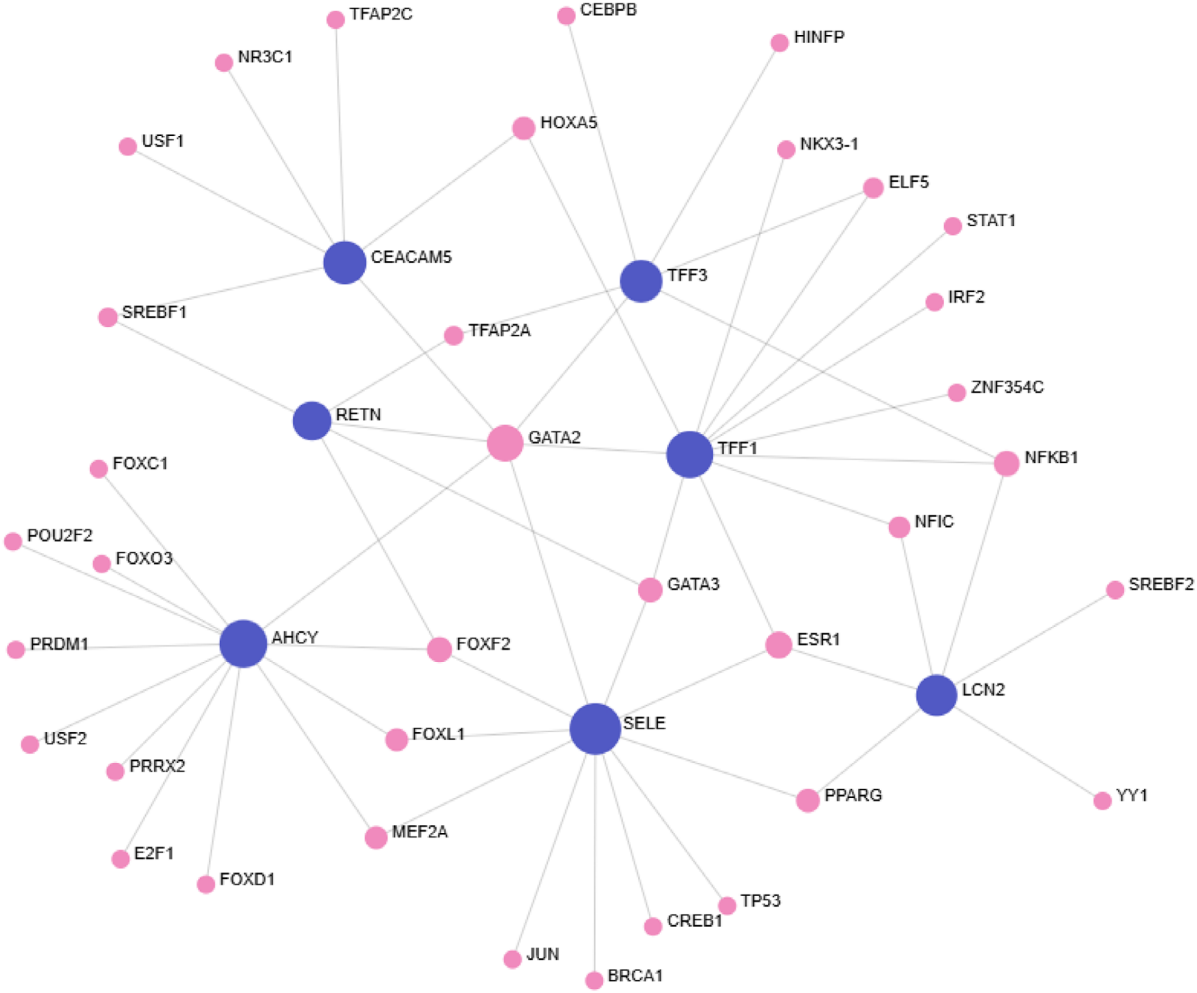
JASPAR transcription factor–protein interaction network. Transcription factor–protein interaction network generated using the JASPAR database for seven CRC‐associated seed proteins (TFF3, TFF1, AHCY, RETN, LCN2, SELE and CEACAM5). All seven proteins were included in a single interconnected network comprising of 34 nodes and 57 edges. AHCY, TFF1 and SELE showed the highest connectivity among the seed proteins. GATA2 was the most connected transcription factor, interacting with six seed proteins and exhibiting greater centrality than several seed nodes. Additional TFs such as ESR1, NF‐κB and FOXF2 also demonstrated moderate connectivity. Dark blue nodes represent seed proteins and pink nodes represent transcription factors. Network visualised using OmicsNet.

JASPAR produced a single 34‐node network with all seven proteins. AHCY, TFF1 and SELE showed the highest centrality (Figure 8). GATA2 was the most connected TF (degree = 6), regulating all seed proteins except LCN2, with higher centrality than some seed nodes. Other notable TFs included ESR1, NF‐κB and FOXF2.

**FIGURE 9:**
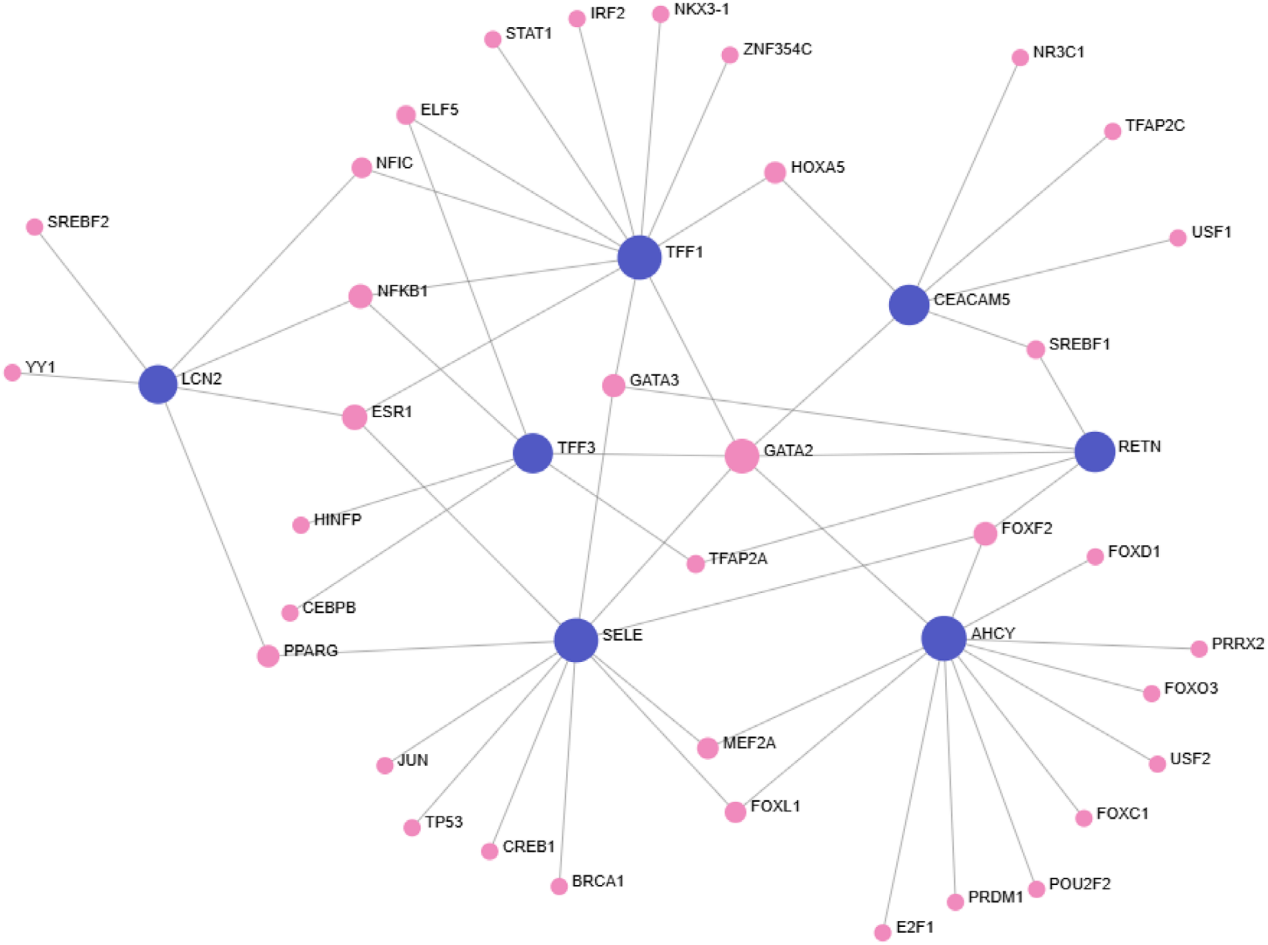
ChEA transcription factor–protein interaction network. Transcription factor–target interaction network generated using the ChEA database for seven CRC‐associated seed proteins (TFF3, TFF1, AHCY, RETN, LCN2, SELE and CEACAM5). The network comprised 120 nodes and 179 edges in a single interconnected network. AHCY, TFF1 and SELE showed the highest regulatory connectivity among seed proteins, whereas LCN2, TFF3, RETN and CEACAM5 exhibited moderate integration. GATA2 was the most connected transcription factor, interacting with six of the seven seed proteins. Other key transcription factors included GATA3, NFkB and ESR1. Dark blue nodes represent seed proteins and pink nodes represent transcription factors. Network visualised using OmicsNet.

ChEA identified 120 nodes and 179 edges. AHCY, TFF1 and SELE again showed the highest centrality (Figure 9). GATA2 was a prominent TF across all proteins except LCN2. RETN remained the least connected.

Overall, AHCY, TFF1 and SELE were the most transcriptionally regulated, while GATA2 and NF‐κB were recurrent key regulators across multiple datasets.

### 2.6 Transcriptomics Validation Using Colonomics Dataset

**FIGURE 10:**
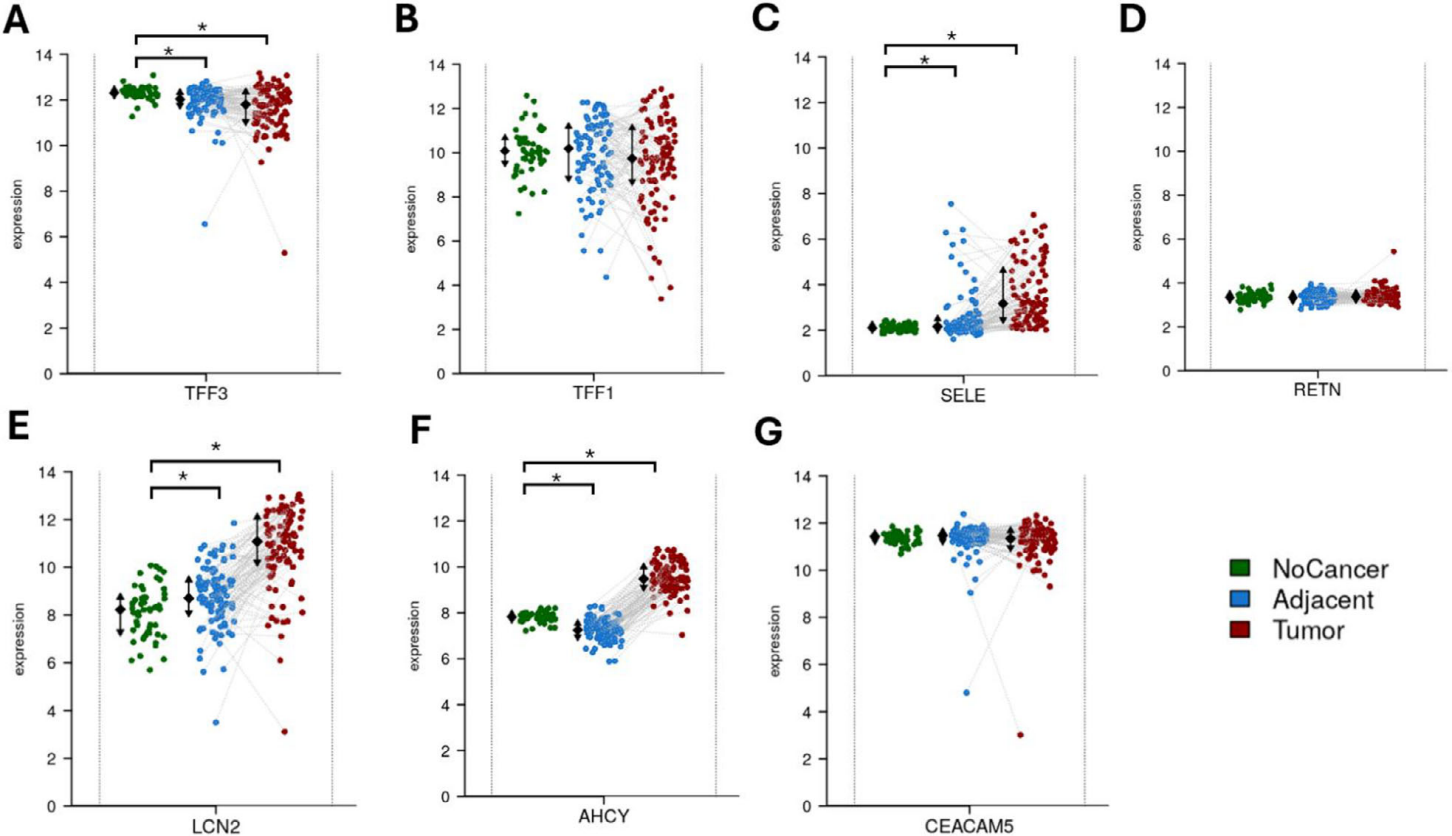
Gene expression of CRC associated proteins across Colonic tissue types in Stage II MSS colon cancer. Transcriptomic expression levels of seven CRC associated genes, (A) TFF3, (B) TFF1, (C) SELE, (D) RETN, (E) AHCY, (F) LCN2 and (G) CEACAM, were analysed using the Colonomics dataset. Expression was assessed across three tissue types, normal mucosa (no cancer, green, *n* = 50), adjacent nontumour tissue (blue, *n* = 98) and tumour tissue (red, *n* = 98). TFF3, SELE, LCN2 and AHCY demonstrated significant differences in tumour and adjacent tissue compared to normal mucosa. Each point represents an individual sample, grey lines connect matched samples from the same patient. Black error bars represent the mean ± (SD) for each group. Visualisation was performed using the Colonomics platform. * *p* < 0.05.

Transcriptomic validation of the seven CRC associated genes (TFF3, TFF1, SELE, RETN, LCN2, AHCY and CEACAM5) was performed using the Colonomics dataset in stage II microsatellite stable (MSS) colon cancer. Expression was analysed across tissue types, gender, molecular subtypes, tumour location and mutation status. TFF3 showed the highest expression in normal mucosa and was significantly downregulated in adjacent and tumour tissues (*p* < 0.001), while TFF1 followed a similar trend but without statistical significance. SELE displayed the lowest expression in normal tissue and was significantly upregulated in tumour samples (*p* < 0.001) (Figure 10). AHCY was downregulated in adjacent tissue but upregulated in tumour tissue (*p* < 0.001), indicating dynamic regulation. LCN2 was significantly elevated in tumour samples, whereas RETN and CEACAM5 showed stable expression with no significant changes. These expression patterns differed from UK Biobank proteomic data, where all seven proteins were upregulated in CRC patients.

**FIGURE 11:**
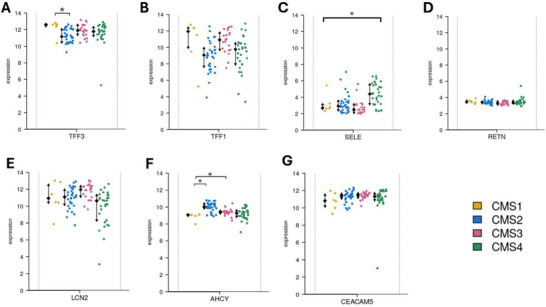
Gene expression of CRC associated proteins across consensus molecular subtypes in Stage II MSS colon cancer tissue. Transcriptomic expression of seven CRC associated proteins, (A) TFF3, (B) TFF1, (C) SELE, (D) RETN, (E) AHCY, (F) LCN2 and (G) CEACAM in tumour tissue samples classified by the four CMS subtypes (CMS1‐4). CMS1 (yellow, *n* = 6), CMS2 (blue, *n* = 31) CMS3 (pink, *n* = 21) and CMS4 (green, *n* = 29). TFF3, SELE and AHCY displayed significant differences between certain CMS groups. Each point represents an individual sample. Black error bars represent the mean ± (SD) for each group. Visualisation was performed using the Colonomics platform. * *p* < 0.05.

No statistically significant differences in gene expression were observed between male and female tumour samples (Supporting Information), suggesting that gender does not strongly influence transcriptional activity for these markers. When grouped by CMS (Figure 11), TFF3 was significantly elevated in CMS1 compared to CMS2 (*p* < 0.05), AHCY was higher in CMS2 and CMS3, and SELE was significantly increased in CMS4. The remaining genes did not show significant variation, implying more general CRC involvement. LCN2 was the only gene with a statistically significant difference based on tumour location, with higher expression in right‐sided tumours (*p* = 0.002). Expression of other genes differed minimally between left and right sides (Supporting Information). Analysis by CIMP revealed no significant expression differences between CIMP‐high and CIMP‐low groups (Supporting Information). Similarly, BRAF V600 (Supporting Information) mutation status did not significantly impact expression of any of the seven genes, however, TFF3, TFF1, SELE and LCN2 showed modest upregulation trends in mutant samples. Finally, KRAS mutation status showed that only SELE expression was significantly reduced in KRAS‐mutant tumours (*p* < 0.05), while the other six genes were unaffected (Supporting Information). Overall, these findings suggest differential regulation of specific CRC‐associated genes across tumour contexts, with notable variation in TFF3, SELE, AHCY, LCN2 and SELE particularly linked to subtype and mutation‐specific processes.

## 3 Discussion

This study aimed to investigate the molecular interplay between CRC and IBD through a multi‐omics approach, with a specific focus on seven previously identified CRC associated proteins (TFF3, TFF1, AHCY, RETN, LCN2, SELE and CEACAM5). The findings of this study verify the hypothesis, revealing significant upregulation of all seven proteins in CRC and six in IBD, then identifying AHCY and LCN2 as central network hubs. Furthermore, the overlap in regulatory nodes, particularly transcription factors such as NF‐κB and GATA2, suggests a shared inflammatory mechanism underlying both conditions. These results reinforce the concept of inflammation driven tumorigenesis and highlight potential associations that bridge chronic inflammation and colorectal carcinogenesis.

### 3.1 Protein Expression and Functional Implications

This study identified significant upregulation of seven CRC associated proteins (TFF3, TFF1, SELE, RETN, AHCY, LCN2 and CEACAM5) in CRC samples compared to controls from the UK Biobank. Furthermore, six of these proteins (except CEACAM5) also displayed significant upregulation in IBD patients in the UK Biobank. The simultaneous upregulation of these proteins in both conditions is consistent with the well‐established link between inflammation and tumorigenesis in the colon [51]. The finding that chronic inflammation drives an increase in pro‐tumorigenic protein supports the hypothesis that IBD associated molecular pathways can underpin the foundation for colorectal tumours.

TFF3 and TFF1 were elevated in CRC, aligning with prior studies that show they are overexpressed in tumour tissues and patient serums [52]. TFF3 has been linked to aggressive cancers as it promotes the epithelial mesenchymal transition and invasion, correlating with poor survival [52]. This suggests that TFF3 in normal conditions protects mucosal integrity but may facilitate cancer progression when chronically overexpressed in inflammatory environments. TFF1's involvement within CRC appears to be more complex. TFF1 has previously been identified as a noninvasive urinary biomarker panel for early CRC detection [53]. However, evidence suggests that TFF1 may play a dual and context dependent role in CRC. Previous studies indicate that TFF1 possesses tumour suppressive properties, as overexpression in CRC cells reduces proliferation, motility and metastasis [54]. One study also reported TFF1 expression was associated with differentiated tumours and tumours lacking metastasis, suggesting TFF1 is linked with less aggressive disease [55]. This suggests that the upregulation could serve as an initial protective response to counteract tumour progression but may become dysfunctional over time, allowing for malignancy to proceed.

LCN2 showed the highest expression out of the proteins in IBD samples and was also significantly elevated in CRC. LCN2 is an innate immune protein that increases during intestinal inflammation, like IBD, and is a biomarker of mucosal injury [56]. The upregulation of LCN2 in both conditions may be a host defence mechanism to maintain inflammation and prevent tumour progression. However, previous studies showed LCN2 decreases in the transition from long term ulcerative colitis to dysplasia contradicting the UK Biobank findings [57]. This inconsistency suggests the role of LCN2 may differ between inflammation driven and sporadic tumorigenesis where it is a protective factor in preneoplastic inflammation that is lost as the dysplasia develops.

CEACAM5 was absent in the IBD dataset despite significant upregulation in CRC samples. CEACAM5 is a well‐established CRC biomarker in clinical use for diagnosis and prognosis. Higher CEACAM5 tumour expression is associated with worse outcomes [58]. CEACAM5's absence in IBD and elevation in CRC supports its specificity to differentiate between tumours and inflammation.

RETN is an adipokine and inflammatory cytokine upregulated in CRC and IBD. This aligns with literature that IBD patients have higher circulating RETN compared to healthy individuals [59]. The upregulation in both conditions further reinforces the inflammatory environment's contribution to tumour biology.

### 3.2 Molecular Interaction Network Analysis

Network analysis revealed how these seven CRC associated proteins interact within broader molecular networks. Through the construction of PPIs, MPIs and transcription factor–protein networks, central hub nodes that may be involved in inflammation and CRC were identified.

AHCY emerged as a central hub across protein–protein, metabolite–protein and transcription factor–protein networks. It demonstrated high connectivity in many networks (degree = 67 in ENCODE) and was the only protein directly interacting with metabolites, highlighting its central role. This importance aligns with AHCY's function associating cellular methylation through hydrolysing *S*‐adenosylohomocysteine (SAH) to adenosine and homocysteine. SAH is a methyltransferase inhibitor. Therefore, its removal promotes DNA, RNA and histone methylation, leading to epigenetic alterations, a key process in tumorigenesis [60]. Further supporting this, AHCY has been found to be upregulated in human CRC tissue and promotes tumour cell proliferation, invasion, angiogenesis and pro‐inflammatory signalling [61]. This, in combination with the upregulation of AHCY, means there is increased AHCY mediated breakdown of SAH, promoting methylation potential. This leads to altered transcriptional activity and epigenetic plasticity, which are hallmarks of cancer [62].

LCN2 displayed multiple connections with inflammatory signalling pathways, like STAT3, NF‐κB and SP1. This supports LCN2's role as a key inflammatory mediator in transcriptional networks. LCN2's overexpression in intestinal epithelial cells promotes inflammation in colitis through triggering pyroptosis and epithelial damage through the NF‐κB/NLRP3/GSDMD pathway, contributing to an environment that supports tumour initiation [63]. It was also found to drive CRC progression through promoting IL‐6/STAT3/NF‐κB signalling, creating a positive feedback loop that sustains both inflammation and cell proliferation in colitis associated cancer models [64]. The interaction between SP1 and LCN2 has been previously demonstrated, with SP1 directly binding to the LCN2 promoter and enhancing its transcription under inflammatory conditions [65]. This transcriptional regulation supports LCN2's role as a functionally integrated proinflammatory mediator in mucosal inflammation.

TFF3 was involved in the transcription factor networks more than protein networks. TFF3 displayed connections to NF‐κB in all transcription factor–protein networks except ENCODE. This interaction has been previously described with TFF3 transiently activating NF‐κB. The transient activation of NF‐κB through TFF3 does not lead to pro‐inflammatory factor induction through the upregulation of TWIST [66]. This suggests that TFF3 creates a feedback loop regulating inflammation, which fits the picture of the inflammatory background of both IBD and CRC.

SELE and TFF1 demonstrated a moderate connection within the network with higher relevance in transcription factor–protein interactions. CEACAM5 showed lower connectivity but still connected within molecular networks; however, RETN was isolated in many of the network models, with them both playing more peripheral roles. This indicates that RETN's regulation is mainly independent of the core networks. This reflects RETN's role as a systemic inflammatory marker rather than a local protein in CRC pathways.

Transcription factor analysis provided further insight into potential regulators linking inflammation and CRC. GATA2 emerged as a prominent transcription factor interacting with all seven proteins, except LCN2, across two networks with high centrality. It regulates angiogenesis, vital for tumour progression and GATA2 overexpression has been associated with advanced CRC and poor outcomes [67]. Mechanistically, GATA2 activates a miR‐31‐mediated pathway that represses SELE, reducing SELE mediated adhesion and migration of colon cancer cells, supporting its potential role as a regulatory hub influencing tumour progression and inflammation‐associated pathways in CRC [68].

Overall, these networks indicate that the proteins upregulated in CRC and IBD are not working in isolation but are involved in complex, integrated pathways with distinct key hubs.

### 3.3 Independent Validation Using the Colonomics Dataset

Validation with the Colonomics dataset assists in the application of the findings. SELE, AHCY and LCN2 were significantly upregulated in colon tumour tissues compared to normal mucosa, mirroring the results of the plasma protein expression, strengthening their relevance as disease linked markers. This agreement reinforces the idea that these proteins are involved in CRC, not just anomalies. However, some proteins displayed discrepancies between systemic and tissue expression. TFF3 expression was downregulated in colon tumour samples and adjacent tissue compared to normal mucosa. This suggests the increased TFF3 in plasma may be due to inflammatory processes or tumour microenvironment rather than the tumour cells themselves. TFF3 demonstrated significantly higher expression in CMS1 compared to the canonical CMS2 subtype. This supports the link between IBD and CRC through CMS1 as the inflammatory subtype displayed the highest expression of TFF3, which is in line with the UK Biobank findings. AHCY demonstrated dynamic expression with significant upregulation in tumour tissue compared to normal mucosa, however, it demonstrated downregulation in adjacent tissue compared to normal mucosa. AHCY was also higher in CMS2 and CM3 compared to CMS1, which fits AHCY's role in metabolism and proliferation. TFF1, RETN or CEACAM5 did not display significant expression differences between tissue types, highlighting that these proteins may not play a significant role in stage II MSS colon cancer. This may be due to the use of stage II samples, as RETN expression is known to increase in later cancer stages [69].

LCN2 was significantly higher in right‐sided tumours compared to left‐side. It has been found right sided CRC tend to experience more immune infiltration [70]. This aligns with previous studies linking LCN2 expression to increased infiltration of CD8+ T cells and macrophages [71]. This proposes that LCN2 may contribute to creating an immunologically active. SELE was higher in CMS4 and the only protein to differ by KRAS mutation status.

None of the proteins showed significant differences by gender, CIMP or BRAF status, inferring that their expression patterns are not restricted to specific molecular subtypes. Further strengthening the relevance of these proteins representing core inflammatory and oncogenic processes rather than subtype specific alterations.

### 3.4 Strengths and Limitations of this Study

This study used robust and strong datasets, each presenting its strengths and limitations [72]. Through integrating proteomics and transcriptomics, it progresses beyond isolated expression profiles to examine proteins in their broader functional context. The use of a large cohort from UK Biobank increases the statistical power and robustness, followed by the independent Colonomics dataset which adds a layer of validation, strengthening the application of the findings. This study went beyond differential expression to explore molecular interaction networks, allowing the identification of functional hubs rather than relying only on expression magnitudes.

Despite this, there are limitations to this study. The UK Biobank data are curated from plasma using Olink's PEA, which reflects systemic expression rather than tissue specific expression. The values are normalised and relative, reducing their ability for direct comparison to gene expression in Colonomics. The Colonomics dataset only includes stage II MSS tumours from 150 patients, meaning other disease states are not represented, such as late stage colon cancer or MSI tumours. The UK Biobank lacks clinical annotations on tumour location, therapy, IBD duration and disease course. This limits the ability to stratify results or to infer disease progression pathways. The IBD dataset lacked CEACAM5 detection and case and control were not matched.

The databases within OmicsNet displayed inconsistencies with the networks produced, complicating the biological interpretation due to data curation differences. The use of Bioinformatic tools offers powerful exploration, which would not be achievable using conventional methods, but often lack experimental validation. Therefore, interactions and pathways are theoretical until confirmed in vivo or in vitro. The network analysis relied on centrality measures to identify central nodes, and while they are beneficial, they do not provide information on causality and the context of the connection.

### 3.5 Future Research

To build upon the findings of the study further research must be performed to enhance the biological relevance and translational potential. Initially, the in silico predictions require experimental biological validation. Key nodes like AHCY and LCN2 should be prioritised for functional studies in CRC models. For example, knockout models of these genes produced through CRISPR‐cas9 or siRNA of CRC cell lines (e.g., HCT116 and SW480) could reveal their roles in cell proliferation, apoptosis and inflammation associated signalling pathways. Overexpression studies of the peripheral proteins, like RETN and CEACAM5, in model systems may demonstrate oncogenic potential that is not obvious from the network alone. These gain and loss of function studies will help to confirm which proteins are drivers and which are passengers of the CRC process.

Second, the generalisability of the results should be evaluated in more diverse populations and cohorts. Both datasets were based on European cohorts, therefore, validating the seven proteins in multi‐ethnic or larger cohorts like The Cancer Genome Atlas would be valuable. This could confirm whether the networks observed can be universally applied or if there are population differences. This approach would also enable subtype analysis, such as investigations into whether these networks can be applied to MSI tumours or colitis associated CRC.

Third, longitudinal data to identify the proteomic changes during the transition from high risk groups, like IBD to CRC. This would help to understand if the proteins upregulated in CRC and IBD are predictors of malignant transformation or if they indicate the inflammatory state.

Finaly, it is important to explicitly distinguish systemic or circulating biomarkers from tissue‐level expression profiles in our interpretation. Circulating protein levels do not necessarily reflect the expression of the corresponding genes in tumour cells or the tumour microenvironment. For example, Trefoil factors themselves are known to be highly tissue‐specific in their normal expression patterns TFF1 predominantly in gastric mucosa and TFF3 in intestinal goblet cells and their dysregulated expression in tumor tissue is well documented across multiple cancers (e.g., increased TFF3 or TFF1 in colorectal and other solid tumors), but these tissue patterns do not necessarily translate to equivalent changes in plasma [73]. Therefore, care should be taken when interpreting plasma proteomic signals as indicators of tumor cell biology; systemic biomarker fluctuations may reflect broader host responses or nontumor cellular contributions.

Future studies should incorporate and adjust for confounders such as age, diet, BMI, smoking status and comorbidities, which all influence inflammation and cancer risk [74]. Adjusting for these parameters will clarify if observed differences are truly due to CRC and IBD and reduce bias.

## 4 Conclusion

This multi‐omics study provides a novel insight into the molecular architecture of CRC and the molecular interplay between chronic inflammation and CRC. The seven associated proteins identified from UK Biobank (TFF3, TFF1, AHCY, RETN, LCN2, SELE and CEACAM5) presented with upregulation in CRC and six of these are also elevated in an inflammatory condition (IBD). AHCY and LCN2 emerged as central hubs with interaction networks, implicating them in key associations involving inflammation, metabolism and epigenetic modification. The concurrent upregulation of six of these proteins in IBD highlights a potential inflammatory bridge between chronic colonic inflammation and malignant transformation, especially through NF‐κB, which was often involved in the networks.

Validation with the Colonomics dataset supported the expression of several proteins, specifically with LCN2, SELE and AHCY, further strengthening their functional relevance. In conclusion, this research strongly supports the hypothesis that inflammatory and oncogenic pathways in the colon are linked through protein interaction networks. These results encourage further experimental validation and open the avenue for developing therapeutic targets for inflammation‐driven colorectal carcinogenesis. The integration of proteomic data with network biology and validation across independent cohorts could be applied to other complex disease intersections, ultimately improving our ability to intervene in CRC development and mechanistic aspects driven by chronic inflammation.

## Author Contributions

J.D. and S.K.R. performed the data analysis and drafted the manuscript. A.A. conceived and designed the study. A.A. supervised and coordinated the study's design. L.B.M. and D.R. helped to get the data from Biobank. SM provided feedback on the analysis. D.R. and A.B.A. provided feedback on biological interpretation. N.D. and R.G. provided feedback on the draft and wrote first draft. All authors revised the manuscript, read and approved the final manuscript.

## Funding

The authors have nothing to report.

## Conflicts of Interest

The authors declare no conflicts of interest.

## Supporting information




**Supporting File 1:** prca70041‐sup‐0001‐SupMat.docx.

## Data Availability

All the data is available online at https://www.colonomics.org/.

## References

[1] F. Bray , M. Laversanne , H. Sung , et al., “Global Cancer Statistics 2022: GLOBOCAN Estimates of Incidence and Mortality Worldwide for 36 Cancers in 185 Countries,” CA: A Cancer Journal for Clinicians 74, no. 3 (2024): 229–263. 38572751 10.3322/caac.21834

[2] M. Arnold , M. S. Sierra , M. Laversanne , I. Soerjomataram , A. Jemal , and F. Bray , “Global Patterns and Trends in Colorectal Cancer Incidence and Mortality,” Gut 66, no. 4 (2016): 683–691, 10.1136/gutjnl-2015-310912. 26818619

[3] J. A. Sninsky , B. M. Shore , G. V. Lupu , and S. D. Crockett , “Risk Factors for Colorectal Polyps and Cancer,” Gastrointestinal Endoscopy Clinics of North America 32, no. 2 (2022): 195–213, https://www.sciencedirect.com/science/article/abs/pii/S1052515721001173?via%3Dihub, [Internet]. 35361331 10.1016/j.giec.2021.12.008

[4] L. H. Nguyen , A. Goel , and D. C. Chung , “Pathways of Colorectal Carcinogenesis,” Gastroenterology 158, no. 2 (2020): 291–302, 10.1053/j.gastro.2019.08.059. 31622622 PMC6981255

[5] B. Leggett and V. Whitehall , “Role of the Serrated Pathway in Colorectal Cancer Pathogenesis,” Gastroenterology 138, no. 6 (2010): 2088–2100, https://www.gastrojournal.org/article/S0016‐5085(10)00172‐1/fulltext, [Internet]. 20420948 10.1053/j.gastro.2009.12.066

[6] R. J. Porter , M. J. Arends , A. M. D. Churchhouse , and S. Din , “Inflammatory Bowel Disease‐Associated Colorectal Cancer: Translational Risks From Mechanisms to Medicines,” Journal of Crohn's and Colitis 15, no. 12 (2021): 2131–2141, 10.1093/ecco-jcc/jjab102. PMC8684457 34111282

[7] T. A. Brentnall , D. A. Crispin , P. S. Rabinovitch , et al., “Mutations in the p53 Gene: An Early Marker of Neoplastic Progression in Ulcerative Colitis,” Gastroenterology 107, no. 2 (1994): 369–378, 10.1016/0016-5085(94)90161-9. 8039614

[8] R. Barna , A. Dema , A. Jurescu , et al., “The Relevance of Sex and Age as Non‐Modifiable Risk Factors in Relation to Clinical‐Pathological Parameters in Colorectal Cancer,” Life 15, no. 2 (2025): 156, https://www.mdpi.com/2075‐1729/15/2/156, [Internet], [cited 2025 Feb 9]. 40003565 10.3390/life15020156 PMC11856218

[9] N. Stjepanovic , L. Moreira , F. Carneiro , et al., “Hereditary Gastrointestinal Cancers: ESMO Clinical Practice Guidelines for Diagnosis, Treatment and Follow‐Up†,” Annals of Oncology 30, no. 10 (2019): 1558–1571, 10.1093/annonc/mdz233. 31378807

[10] P. Rawla , T. Sunkara , and A. Barsouk , “Epidemiology of Colorectal Cancer: Incidence, Mortality, Survival, and Risk Factors,” Gastroenterology Review 14, no. 2 (2019): 89–103, https://pmc.ncbi.nlm.nih.gov/articles/PMC6791134/. 31616522 10.5114/pg.2018.81072 PMC6791134

[11] J. Cheng , Y. Chen , X. Wang , et al., “Meta‐Analysis of Prospective Cohort Studies of Cigarette Smoking and the Incidence of Colon and Rectal Cancers,” European Journal of Cancer Prevention 24, no. 1 (2015): 6–15, 10.1097/CEJ.0000000000000011. 24722538

[12] V. Fedirko , I. Tramacere , V. Bagnardi , et al., “Alcohol Drinking and Colorectal Cancer Risk: An Overall and Dose–response Meta‐Analysis of Published Studies,” Annals of Oncology 22, no. 9 (2011): 1958–1972, https://www.sciencedirect.com/science/article/pii/S0923753419383425, [Internet]. 21307158 10.1093/annonc/mdq653

[13] K. E. Bradbury , N. Murphy , and T. J. Key , “Diet and Colorectal Cancer in UK Biobank: A Prospective Study,” International Journal of Epidemiology 49, no. 1 (2019): 246–258, https://www.ncbi.nlm.nih.gov/pmc/articles/PMC7124508/. 10.1093/ije/dyz064 PMC7124508 30993317

[14] R. J. Turesky , “Mechanistic Evidence for Red Meat and Processed Meat Intake and Cancer Risk: A Follow‐Up on the International Agency for Research on Cancer Evaluation of 2015,” CHIMIA International Journal for Chemistry 72, no. 10 (2018): 718–724., 10.2533/chimia.2018.718. PMC6294997 30376922

[15] IARC Working Group on the Evaluation of Carcinogenic Risk to Humans . Red Meat and Processed Meat [Internet] (Nih.gov. International Agency for Research on Cancer, 2018), https://www.ncbi.nlm.nih.gov/books/NBK507971/. 29949327

[16] H. Oh , H. Kim , D. H. Lee , et al., “Different Dietary Fibre Sources and Risks of Colorectal Cancer and Adenoma: A Dose–response Meta‐Analysis of Prospective Studies,” British Journal of Nutrition 122, no. 6 (2019): 605–615, 10.1017/S0007114519001454. 31495339

[17] N. Papadimitriou , N. Dimou , K. K. Tsilidis , et al., “Physical Activity and Risks of Breast and Colorectal Cancer: A Mendelian Randomisation Analysis,” Nature Communications 11, no. 1 (2020): 597, https://www.nature.com/articles/s41467‐020‐14389‐8, [Internet], [cited 2021 Feb 23]. 10.1038/s41467-020-14389-8 PMC6992637 32001714

[18] D. P. Shah , A. Di Re , and J. Wei , “Aspirin Chemoprevention in Colorectal Cancer: Network Meta‐Analysis of Low, Moderate, and High Doses,” British Journal of Surgery 110, no. 12 (2023): 1691–1702, 10.1093/bjs/znad231. 37499126

[19] E. Wieten , E. H. Schreuders , E. J. Grobbee , et al., “Incidence of Faecal Occult Blood Test Interval Cancers in Population‐Based Colorectal Cancer Screening: A Systematic Review and Meta‐Analysis,” Gut 68, no. 5 (2018): 873–881, 10.1136/gutjnl-2017-315340. 29934436

[20] NHS . Overview—Bowel Cancer Screening [Internet], [cited 2025 Feb 15] (NHS, 2021), https://www.nhs.uk/conditions/bowel‐cancer‐screening/.

[21] Health . Fingertips | Department of Health and Social Care, [Internet] (Phe.org.uk, 2025), https://fingertips.phe.org.uk/search/cancer%20screening#page/4/gid/1938133365/pat/159/par/K02000001/ati/15/are/E92000001/iid/92600/age/280/sex/4/cat/‐1/ctp/‐1/yrr/1/cid/4/tbm/1.

[22] M. Araghi , I. Soerjomataram , A. Bardot , et al., “Changes in Colorectal Cancer Incidence in Seven High‐Income Countries: A Population‐Based Study,” Lancet Gastroenterology & Hepatology 4, no. 7 (2019): 511–518, https://www.thelancet.com/journals/langas/article/PIIS2468‐1253(19)30147‐5/fulltext?hss_channel=tw‐27013292, [Internet]. 31105047 10.1016/S2468-1253(19)30147-5 PMC7617144

[23] S. A. Stapley , G. P. Rubin , D. Alsina , E. A. Shephard , M. D. Rutter , and W. T. Hamilton , “Clinical Features of Bowel Disease in Patients Aged <50 Years in Primary Care: A Large Case‐Control Study,” British Journal of General Practice 67, no. 658 (2017): e336–e344. 10.3399/bjgp17X690425 PMC5409433 28347985

[24] M. Lutgens , M. G. H. van Oijen , G. van der Heijden , F. P. Vleggaar , P. D. Siersema , and B. Oldenburg , “Declining Risk of Colorectal Cancer in Inflammatory Bowel Disease: An Updated Meta‐Analysis of Population‐Based Cohort Studies,” Inflammatory Bowel Diseases 19, no. 4 (2013): 789–799, https://pubmed.ncbi.nlm.nih.gov/23448792/, [Internet], [cited 2020 Jul 12]. 23448792 10.1097/MIB.0b013e31828029c0

[25] C. McDowell , U. Farooq , and M. Haseeb , “Inflammatory Bowel Disease (IBD) [Internet],” in PubMed (StatPearls Publishing, 2023), https://www.ncbi.nlm.nih.gov/books/NBK470312/. 29262182

[26] R. Wang , Z. Li , S. Liu , and D. Zhang , “Global, Regional and National Burden of Inflammatory Bowel Disease in 204 Countries and territories From 1990 to 2019: A Systematic Analysis Based on the Global Burden of Disease Study 2019,” BMJ Open 13, no. 3 (2023): 065186, https://bmjopen.bmj.com/content/13/3/e065186, [Internet]. 10.1136/bmjopen-2022-065186 PMC10069527 36977543

[27] C. D. Gillen , R. S. Walmsley , P. Prior , H. A. Andrews , and R. N. Allan , “Ulcerative Colitis and Crohn's Disease: A Comparison of the Colorectal Cancer Risk in Extensive Colitis,” Gut 35, no. 11 (1994): 1590–1592, 10.1136/gut.35.11.1590. 7828978 PMC1375617

[28] Y. Hasin , M. Seldin , and A. Lusis , “Multi‐Omics Approaches to Disease,” Genome Biology 18, no. 1 (2017): 83, https://genomebiology.biomedcentral.com/articles/10.1186/s13059‐017‐1215‐1. 28476144 10.1186/s13059-017-1215-1 PMC5418815

[29] S. Chakraborty , G. Sharma , S. Karmakar , and S. Banerjee , “Multi‐OMICS Approaches in Cancer Biology: New Era in Cancer Therapy,” Biochimica et Biophysica Acta (BBA)—Molecular Basis of Disease 1870, no. 5 (2024): 167120, 10.1016/j.bbadis.2024.167120. 38484941

[30] M. Du , D. Gu , J. Xin , et al., “Integrated Multi‐Omics Approach to Distinct Molecular Characterization and Classification of Early‐Onset Colorectal Cancer,” Cell Reports Medicine 4, no. 3 (2023): 100974, 10.1016/j.xcrm.2023.100974. 36921601 PMC10040411

[31] L. Xie , Q. Kong , M. Ai , et al., “Spatial Proteomic Profiling of Colorectal Cancer Revealed Its Tumor Microenvironment Heterogeneity,” Journal of Proteome Research 23, no. 8 (2024): 3342–3352, 10.1021/acs.jproteome.3c00719. 39026393

[32] M. Cui , C. Cheng , and L. Zhang , “High‐Throughput Proteomics: A Methodological Mini‐Review,” Laboratory Investigation; A Journal of Technical Methods and Pathology 102, no. 11 (2022): 1170–1181, https://pubmed.ncbi.nlm.nih.gov/35922478/. 35922478 10.1038/s41374-022-00830-7 PMC9362039

[33] J. Guinney , R. Dienstmann , X. Wang , et al., “The Consensus Molecular Subtypes of Colorectal Cancer,” Nature Medicine 21, no. 11 (2015): 1350–1356, 10.1038/nm.3967. PMC4636487 26457759

[34] Z. Liu , S. Weng , Q. Dang , et al., “Gene Interaction Perturbation Network Deciphers a High‐Resolution Taxonomy in Colorectal Cancer,” eLife [Internet] 11 (2022): 81114, https://pubmed.ncbi.nlm.nih.gov/36345721/. 10.7554/eLife.81114 PMC9643007 36345721

[35] S. K. Radhakrishnan , D. Nath , D. Russ , et al., “Machine Learning‐Based Identification of Proteomic Markers in Colorectal Cancer Using UK Biobank Data,” Frontiers in Oncology 14 (2025): 1505675. 39839775 10.3389/fonc.2024.1505675 PMC11746037

[36] C. Sudlow , J. Gallacher , N. Allen , et al., “UK Biobank: An Open Access Resource for Identifying the Causes of a Wide Range of Complex Diseases of Middle and Old Age,” PLOS Medicine 12, no. 3 (2015): 1001779, 10.1371/journal.pmed.1001779. PMC4380465 25826379

[37] G. Zhou , Z. Pang , Y. Lu , J. Ewald , and J. Xia , “OmicsNet 2.0: A Web‐Based Platform for Multi‐Omics Integration and Network Visual Analytics,” Nucleic Acids Research 50, no. W1 (2022): W527–W533, 10.1093/nar/gkac376. 35639733 PMC9252810

[38] K. Breuer , A. K. Foroushani , M. R. Laird , et al., “InnateDB: Systems Biology of Innate Immunity and Beyond—Recent Updates and Continuing Curation,” Nucleic Acids Research 41, no. D1 (2012): D1228–D1233, 10.1093/nar/gks1147. 23180781 PMC3531080

[39] D. Szklarczyk , R. Kirsch , M. Koutrouli , et al., “The STRING Database in 2023: Protein–protein Association Networks and Functional Enrichment Analyses for any Sequenced Genome of Interest,” Nucleic Acids Research 51, no. D1 (2022): D638–D646, https://pubmed.ncbi.nlm.nih.gov/36370105/. 10.1093/nar/gkac1000 PMC9825434 36370105

[40] S. Orchard , M. Ammari , B. Aranda , et al., “The MIntAct Project—IntAct as a Common Curation Platform for 11 Molecular Interaction Databases,” Nucleic Acids Research 42, no. D1 (2013): D358–D363, https://academic.oup.com/nar/article/42/D1/D358/1051282. 24234451 10.1093/nar/gkt1115 PMC3965093

[41] K. Luck , D. K. Kim , L. Lambourne , et al., “A Reference Map of the human Binary Protein Interactome,” Nature 580, no. 7803 (2020): 402–408, https://www.ncbi.nlm.nih.gov/pmc/articles/PMC7169983/, [Internet]. 32296183 10.1038/s41586-020-2188-x PMC7169983

[42] M. Kanehisa , M. Furumichi , M. Tanabe , Y. Sato , and K. Morishima , “KEGG: New Perspectives on Genomes, Pathways, Diseases and Drugs,” Nucleic acids research 45, no. D1 (2017): D353–D361, https://www.ncbi.nlm.nih.gov/pubmed/27899662. 27899662 10.1093/nar/gkw1092 PMC5210567

[43] E. Brunk , S. Sahoo , D. C. Zielinski , et al., “Recon3D enables a Three‐Dimensional View of Gene Variation in human Metabolism,” Nature Biotechnology 36, no. 3 (2018): 272–281, 10.1038/nbt.4072. PMC5840010 29457794

[44] H. Han , J. W. Cho , S. Lee , et al., “TRRUST v2: An Expanded Reference Database of human and Mouse Transcriptional Regulatory Interactions,” Nucleic Acids Research 46, no. D1 (2017): D380–D386, 10.1093/nar/gkx1013. PMC5753191 29087512

[45] M. P. Snyder , T. R. Gingeras , J. E. Moore , et al., “Perspectives on Encode,” Nature 583, no. 7818 (2020): 693–698. 32728248 10.1038/s41586-020-2449-8 PMC7410827

[46] J. A. Castro‐Mondragon , R. Riudavets‐Puig , I. Rauluseviciute , et al., “JASPAR 2022: The 9th Release of the Open‐Access Database of Transcription Factor Binding Profiles,” Nucleic Acids Research 50, no. D1 (2021): D165–D173, 10.1093/nar/gkab1113. PMC8728201 34850907

[47] A. B. Keenan , D. Torre , A. Lachmann , et al., “ChEA3: Transcription Factor Enrichment Analysis by Orthogonal Omics Integration,” Nucleic Acids Research 47, no. W1 (2019): W212–W224, https://academic.oup.com/nar/article/47/W1/W212/5494769, [Internet]. 31114921 10.1093/nar/gkz446 PMC6602523

[48] M. Wang , H. Wang , and H. A. Zheng , “Mini Review of Node Centrality Metrics in Biological Networks,” International Journal of Network Dynamics and Intelligence (2022): 99–110, 10.53941/ijndi0101009.

[49] A. Díez‐Villanueva , R. Sanz‐Pamplona , X. Solé , et al., “COLONOMICS—integrative Omics Data of One Hundred Paired Normal‐Tumoral Samples From Colon Cancer Patients,” Scientific data 9, no. 1 (2022): 595, https://pubmed.ncbi.nlm.nih.gov/36182938/, [Internet]. 36182938 10.1038/s41597-022-01697-5 PMC9526730

[50] S. G. Kwak and J. H. Kim , “Central Limit Theorem: The Cornerstone of Modern Statistics,” Korean Journal of Anesthesiology 70, no. 2 (2017): 144–156, https://pmc.ncbi.nlm.nih.gov/articles/PMC5370305/, [Internet]. 28367284 10.4097/kjae.2017.70.2.144 PMC5370305

[51] I. Mignini , M. E. Ainora , S. D. Francesco , L. Galasso , A. Gasbarrini , and M. A. Zocco , “Tumorigenesis in Inflammatory Bowel Disease: Microbiota‐Environment Interconnections,” Cancers 15, no. 12 (2023): 3200, 10.3390/cancers15123200. 37370812 PMC10295963

[52] A. Yusufu , P. Shayimu , R. Tuerdi , C. Fang , F. Wang , and H. Wang , “TFF3 and TFF1 Expression Levels Are Elevated in Colorectal Cancer and Promote the Malignant Behavior of Colon Cancer by Activating the EMT Process,” International Journal of Oncology 55, no. 4 (2019): 789–804, https://www.ncbi.nlm.nih.gov/pmc/articles/PMC6741840/, [Internet], [cited 2020 Apr 14]. 31432157 10.3892/ijo.2019.4854 PMC6741840

[53] Y. Okuda , T. Shimura , Y. Abe , et al., “Urinary Dipeptidase 1 and Trefoil Factor 1 Are Promising Biomarkers for Early Diagnosis of Colorectal Cancer,” Journal of Gastroenterology 59, no. 7 (2024): 572–585, 10.1007/s00535-024-02110-1. 38836911

[54] A. Saha , N. Gavert , T. Brabletz , and A. Ben‐Ze'ev , “Downregulation of the Tumor Suppressor TFF1 Is Required During Induction of Colon Cancer Progression by L1,” Cancers [Internet] 14, no. 18 (2022): 4478, https://pubmed.ncbi.nlm.nih.gov/36139637/, Autumn. 36139637 10.3390/cancers14184478 PMC9497096

[55] A. Yusup , B. Huji , C. Fang , et al., “Expression of Trefoil Factors and TWIST1 in Colorectal Cancer and Their Correlation With Metastatic Potential and Prognosis,” World Journal of Gastroenterology 23, no. 1 (2017): 110–120, https://www.ncbi.nlm.nih.gov/pmc/articles/PMC5221274/pdf/WJG‐23‐110.pdf, [cited 2020 Jun 1]. 28104986 10.3748/wjg.v23.i1.110 PMC5221274

[56] X. Xiao , B. S. Yeoh , and M. Vijay‐Kumar , “Lipocalin 2: An Emerging Player in Iron Homeostasis and Inflammation,” Annual Review of Nutrition 37, no. 1 (2017): 103–130. 10.1146/annurev-nutr-071816-064559 28628361

[57] F. Kou , Y. Cheng , L. Shi , et al., “LCN2 as a Potential Diagnostic Biomarker for Ulcerative Colitis‐Associated Carcinogenesis Related to Disease Duration,” Frontiers in Oncology 11 (2022): 793760, https://www.ncbi.nlm.nih.gov/pmc/articles/PMC8801604/, [cited 2024 Feb 23]. 35111677 10.3389/fonc.2021.793760 PMC8801604

[58] S. C. Huang , S. C. Chang , T. T. Liao , and M. H. Yang , “Detection and Clinical Significance of CEACAM5 Methylation in Colorectal Cancer Patients,” Cancer Science 115, no. 1 (2024): 270–282, https://pubmed.ncbi.nlm.nih.gov/37942534/. 37942534 10.1111/cas.16012 PMC10823287

[59] A. H. Behnoush , S. P. Maroufi , T. Reshadmanesh , et al., “Circulatory Resistin Levels in Inflammatory Bowel Disease: A Systematic Review and Meta‐Analysis,” BMC Gastroenterology 24 (2024): 107, https://www.ncbi.nlm.nih.gov/pmc/articles/PMC10941394/, [Internet]. 38486190 10.1186/s12876-024-03199-7 PMC10941394

[60] P. Vizán , L. Di Croce , and S. Aranda , “Functional and Pathological Roles of AHCY,” Frontiers in Cell and Developmental Biology 9 (2021): 654344. 33869213 10.3389/fcell.2021.654344 PMC8044520

[61] J. Wang , K. Ding , and Y. Wang , “Wumei Pill Ameliorates AOM/DSS‐Induced Colitis‐Associated Colon Cancer Through Inhibition of Inflammation and Oxidative Stress by Regulating S‐Adenosylhomocysteine Hydrolase‐ (AHCY‐) Mediated Hedgehog Signaling in Mice,” Oxidative Medicine and Cellular Longevity 2022 (2022): 4061713. 35927991 10.1155/2022/4061713 PMC9345734

[62] D. Hanahan , “Hallmarks of Cancer: New Dimensions,” Cancer Discovery 12, no. 1 (2022): 31–46, 10.1158/2159-8290.CD-21-1059. 35022204

[63] Y. Yang , S. Li , K. Liu , et al., “Lipocalin‐2‐mediated Intestinal Epithelial Cells Pyroptosis via NF‐κB/NLRP3/GSDMD Signaling Axis Adversely Affects Inflammation in Colitis,” Biochimica et biophysica acta Molecular Basis of Disease 1870, no. 7 (2024): 167279, https://pubmed.ncbi.nlm.nih.gov/38844113/. 38844113 10.1016/j.bbadis.2024.167279

[64] S. L. Kim , M. W. Shin , S. Y. Seo , and S. W. Kim , “Lipocalin 2 Potentially Contributes to Tumorigenesis From Colitis via IL‐6/STAT3/NF‐κB Signaling Pathway,” Bioscience Reports 42, no. 5 (2022): BSR20212418, https://pubmed.ncbi.nlm.nih.gov/35470375/, [Internet], [cited 2023 Mar 27]. 35470375 10.1042/BSR20212418 PMC9109459

[65] Z. Wang , X. Zhou , X. Hu , and C. Zheng , “Quercetin Ameliorates Helicobacter Pylori‐Induced Gastric Epithelial Cell Injury by Regulating Specificity Protein 1/Lipocalin 2 Axis in Gastritis,” Journal of Applied Toxicology: JAT 44, no. 4 (2024): 641–650, https://pubmed.ncbi.nlm.nih.gov/38056887/. 38056887 10.1002/jat.4566

[66] Y. Q. Zhu and X. D. Tan , “TFF3 modulates NF‐κB and a Novel Negative Regulatory Molecule of NF‐κB in Intestinal Epithelial Cells via a Mechanism Distinct From TNF‐α,” American Journal of Physiology‐Cell Physiology 289, no. 5 (2005): C1085–C1093, 10.1152/ajpcell.00185.2005. 16014704 PMC2527239

[67] L. Chen , B. Jiang , Z. Wang , et al., “Expression and Prognostic Significance of GATA‐Binding Protein 2 in Colorectal Cancer,” Medical Oncology 30, no. 2 (2013): 498, 10.1007/s12032-013-0498-7. 23423786

[68] L. Zhong , J. Huot , and M. J. Simard , “p38 activation Induces Production of miR‐146a and miR‐31 to Repress E‐Selectin Expression and Inhibit Transendothelial Migration of Colon Cancer Cells,” Scientific Reports 8, no. 1 (2018): 2334, 10.1038/s41598-018-20837-9. 29402939 PMC5799178

[69] T. Eguchi Nakajima , Y. Yamada , T. Hamano , et al., “Adipocytokines as New Promising Markers of Colorectal Tumors: Adiponectin for Colorectal Adenoma, and Resistin and Visfatin for Colorectal Cancer,” Cancer Science 101, no. 5 (2010): 1286–1291, 10.1111/j.1349-7006.2010.01518.x. 20331631 PMC11159666

[70] Y. Hu , J. Ding , C. Wu , et al., “Differential Expression and Prognostic Correlation of Immune Related Factors between Right and Left Side Colorectal Cancer,” Frontiers in Oncology 12 (2022): 845765. 35936748 10.3389/fonc.2022.845765 PMC9353740

[71] W. X. Xu , J. Zhang , Y. T. Hua , S. J. Yang , D. D. Wang , and J. H. Tang , “An Integrative Pan‐Cancer Analysis Revealing LCN2 as an Oncogenic Immune Protein in Tumor Microenvironment,” Frontiers in Oncology 10 (2020): 605097, https://www.ncbi.nlm.nih.gov/pmc/articles/PMC7786136/, [Internet], [cited 2022 Oct 25]. 33425761 10.3389/fonc.2020.605097 PMC7786136

[72] M. Conroy , J. Sellors , M. Effingham , et al., “The Advantages of UK Biobank's Open‐Access Strategy for Health Research,” Journal of Internal Medicine 286, no. 4 (2019): 389–397, https://onlinelibrary.wiley.com/doi/10.1111/joim.12955, [Internet]. 31283063 10.1111/joim.12955 PMC6790705

[73] Y. Zhang , Y. Liu , L. Wang , and H. Song , “The Expression and Role of Trefoil Factors in human Tumors,” Translational Cancer Research 8, no. 4 (2019): 1609–1617, 10.21037/tcr.2019.07.48. 35116904 PMC8798463

[74] A. Skelly , J. Dettori , and E. Brodt , “Assessing Bias: The Importance of Considering Confounding,” Evidence‐Based Spine‐Care Journal 3, no. 1 (2012): 9–12, https://pmc.ncbi.nlm.nih.gov/articles/PMC3503514/, [Internet]. 10.1055/s-0031-1298595 PMC3503514 23236300

