# Supplementary material for: A Network‐Based Association of IBD and Colorectal Cancer Using Proteomics Data
Source: Proteomics Clin Appl. 2026 Feb 17;20(2):e70041. doi: 10.1002/prca.70041 (PMC12914161; doi:10.1002/prca.70041)
Supplement: Supplementary file 1 — Supporting File 1: prca70041‐sup‐0001‐SupMat.docx. [file PRCA-20-e70041-s001.docx]

**Supplementary files**

**A Network-Based Association of IBD and Colorectal Cancer Using Proteomics Data**

Jaiya Dhani ^1,#^, Swarnima Kollampallath Radhakrishnan^1,#^, Dominic Russ^1,2,3^, Sudip Mondal^1,2,3^, Abdulrahman Alzarooni^1,2,3^, Laura Bravo Merodio^1,2,3^, Niharika A Duggal^4,5^, Ruchi Gupta^6^, Animesh Acharjee^1,2,3*^

^1^Cancer and Genomic Sciences, School of Medical Sciences, College of Medicine and Health, University of Birmingham, UK

^2^Centre for Health Data Research, University of Birmingham, Birmingham, UK

^3^Institute of Translational Medicine, University Hospitals Birmingham NHS, Foundation Trust, UK

^4^MRC-Versus Arthritis Centre for Musculoskeletal Ageing Research

^5^Institute of Inflammation and Ageing, University of Birmingham, Birmingham B15 2TT, United Kingdom

^6^School of Chemistry, University of Birmingham, Birmingham, UK

**# Equal contribution**

***Correspondence**

Dr. Animesh Acharjee

University of Birmingham, B15 2TT, UK

E-mail: [a.acharjee@bham.ac.uk](mailto:a.acharjee@bham.ac.uk)

Phone: +44 121 414 7012


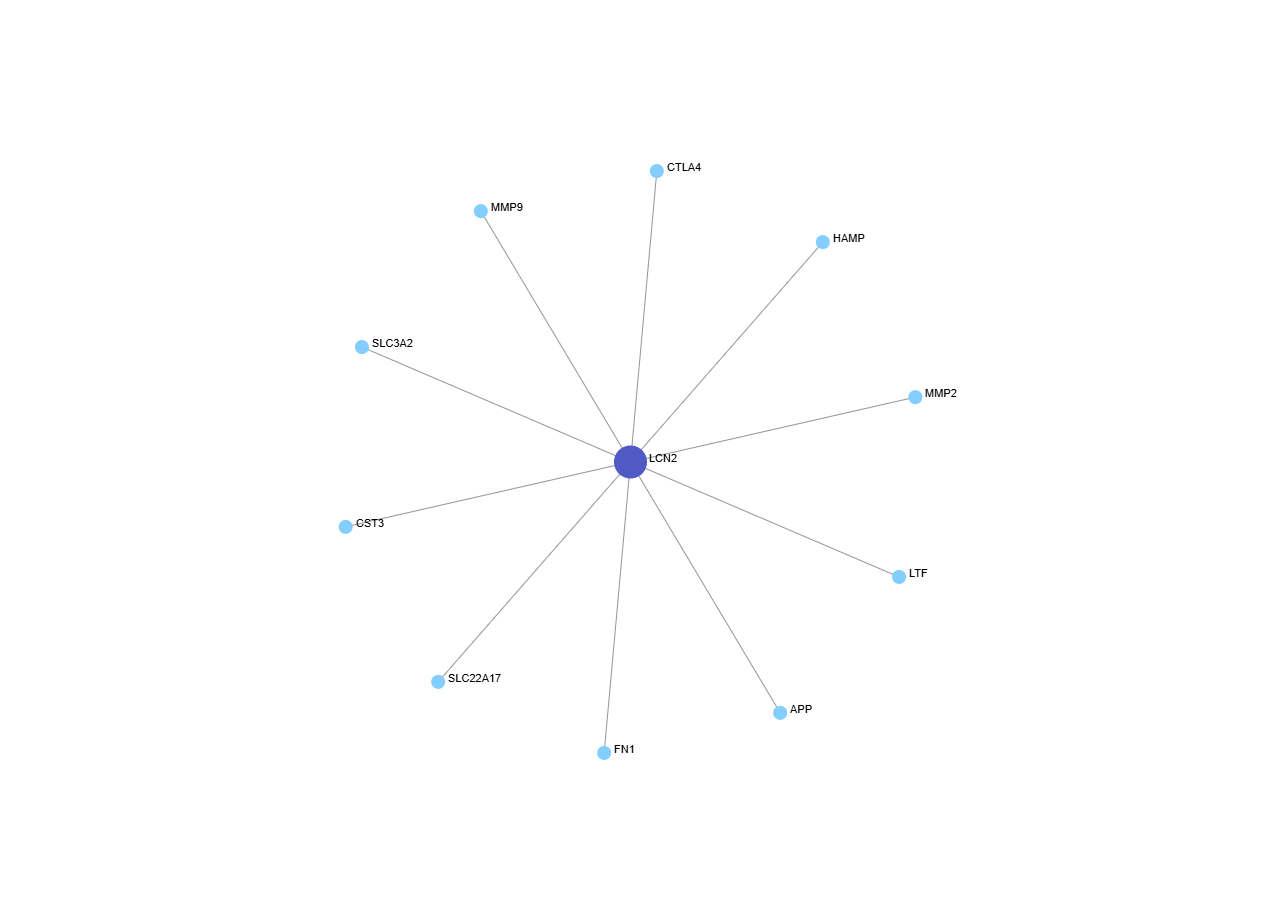

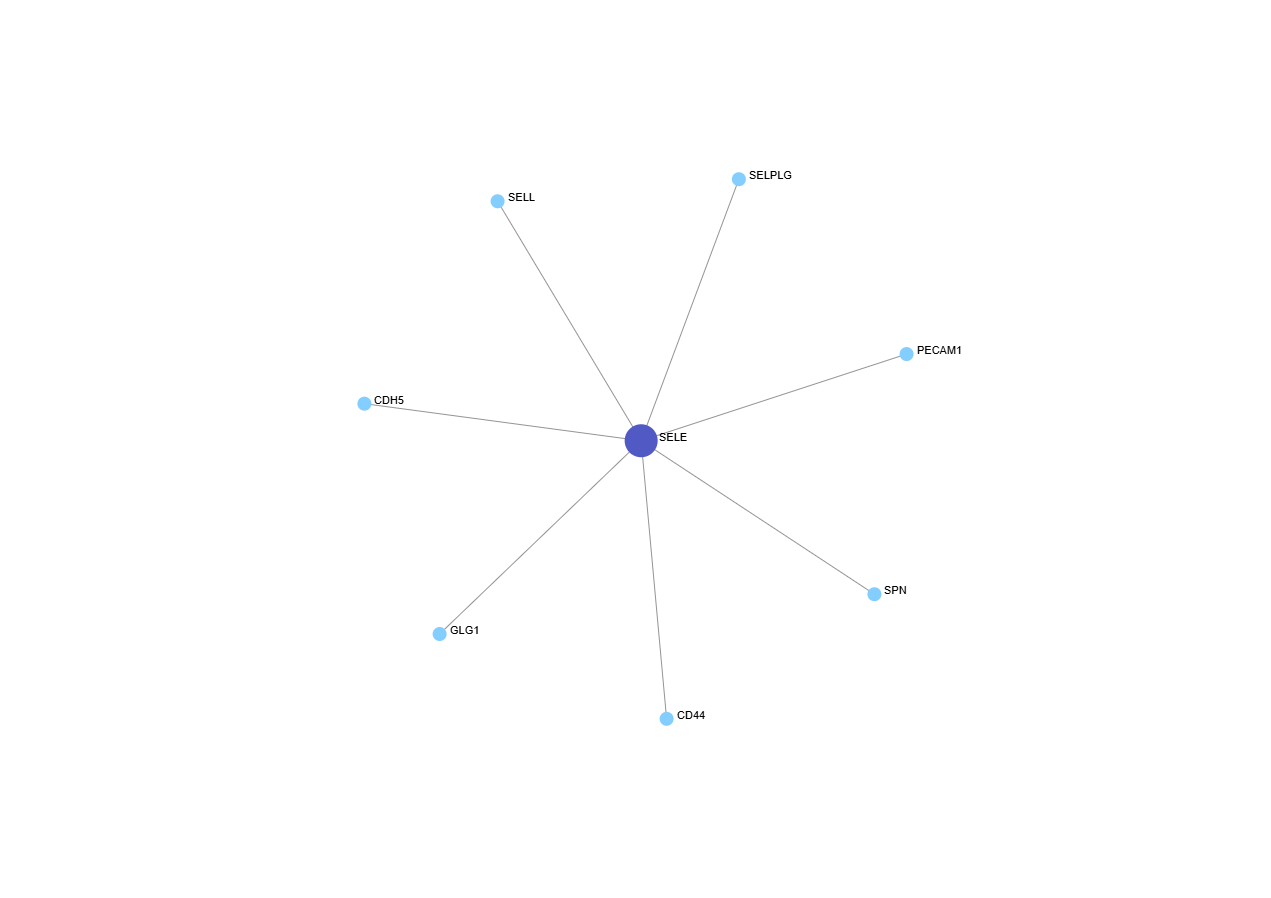

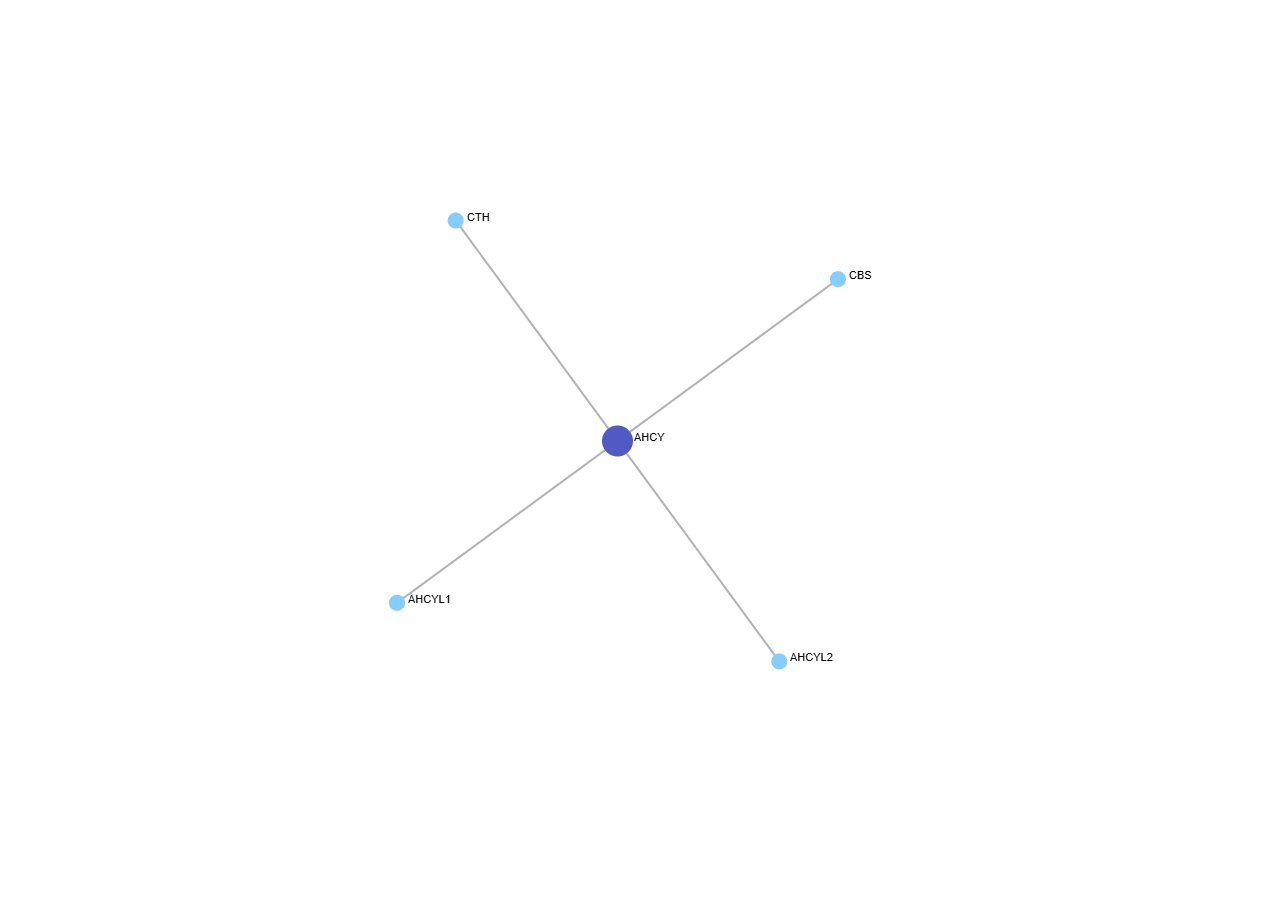

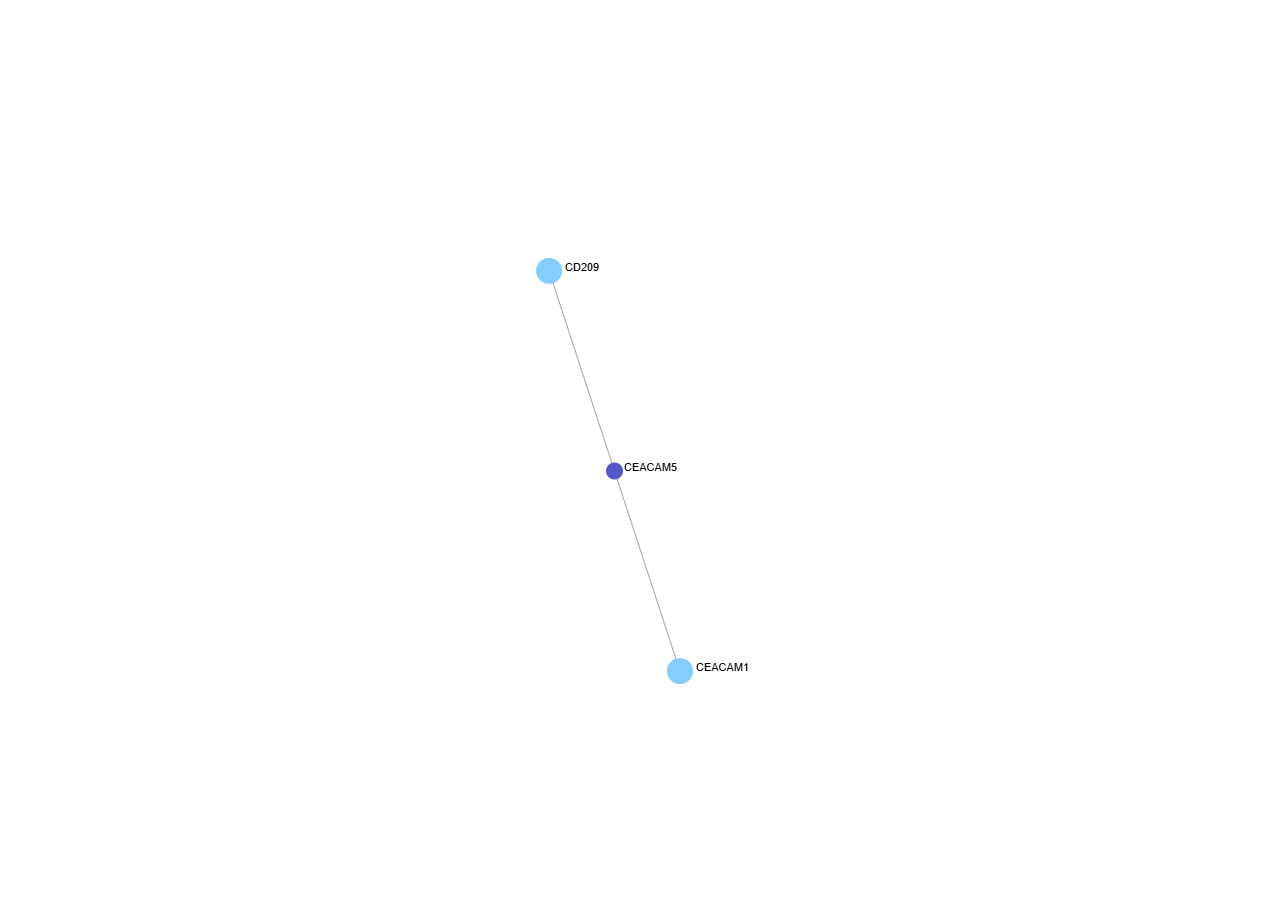

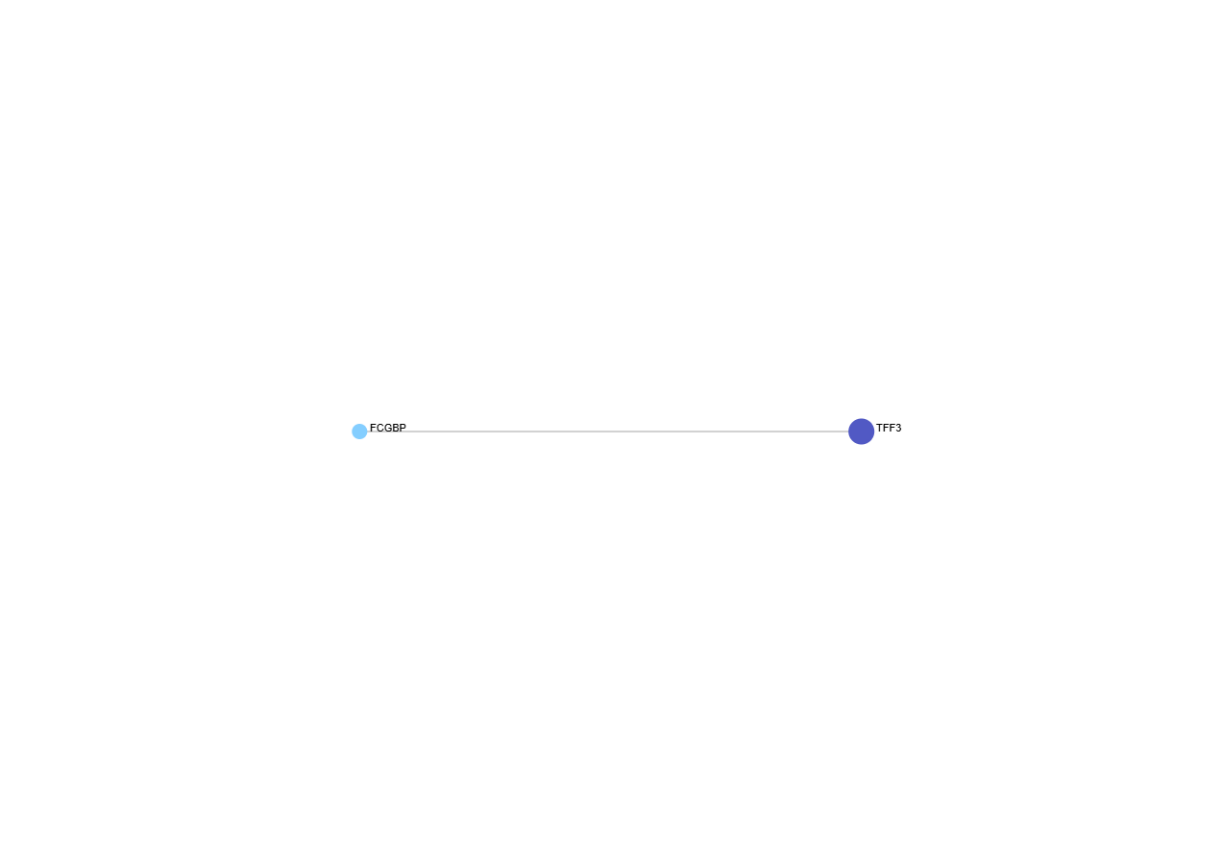

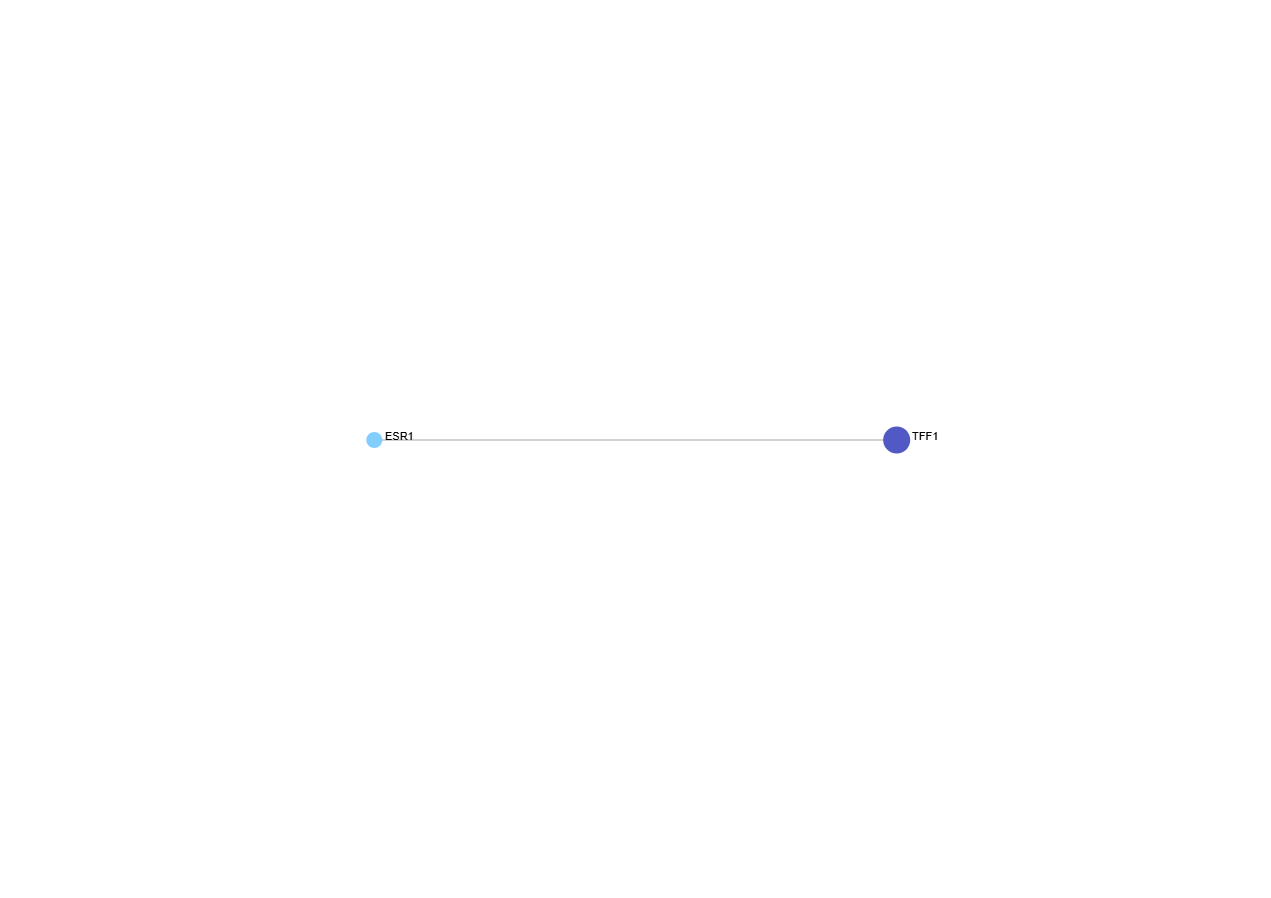


**A**

**C**

**D**

**E**

**B**

**F**

***Supplementary Figure 1: STRING PPI Network***

*PPI network generated using STRING for seven CRC associated seed proteins (TFF3, TFF1, AHCY, RETN, SELE, LCN2, CEACAM5). The network includes six of the seven proteins, RETN was not included. The network is highly fragmented, with each seed protein in its own independent subnetwork. Across these six subnetworks from the six seed proteins, there are 31 nodes connected by 50 edges. (A) LCN2 (nodes = 11, edges =10), (B) SELE, (C) AHCY, (D) CEACAM5, (E) TFF1 and (F) TFF3. Each seed protein is only connected to its direct interaction, with no cross seed interactions observed. Dark blue nodes indicate seed proteins, light blue nodes represent proteins. Grey edges represent direct protein-protein interactions. Network visualised using OmicsNet.*


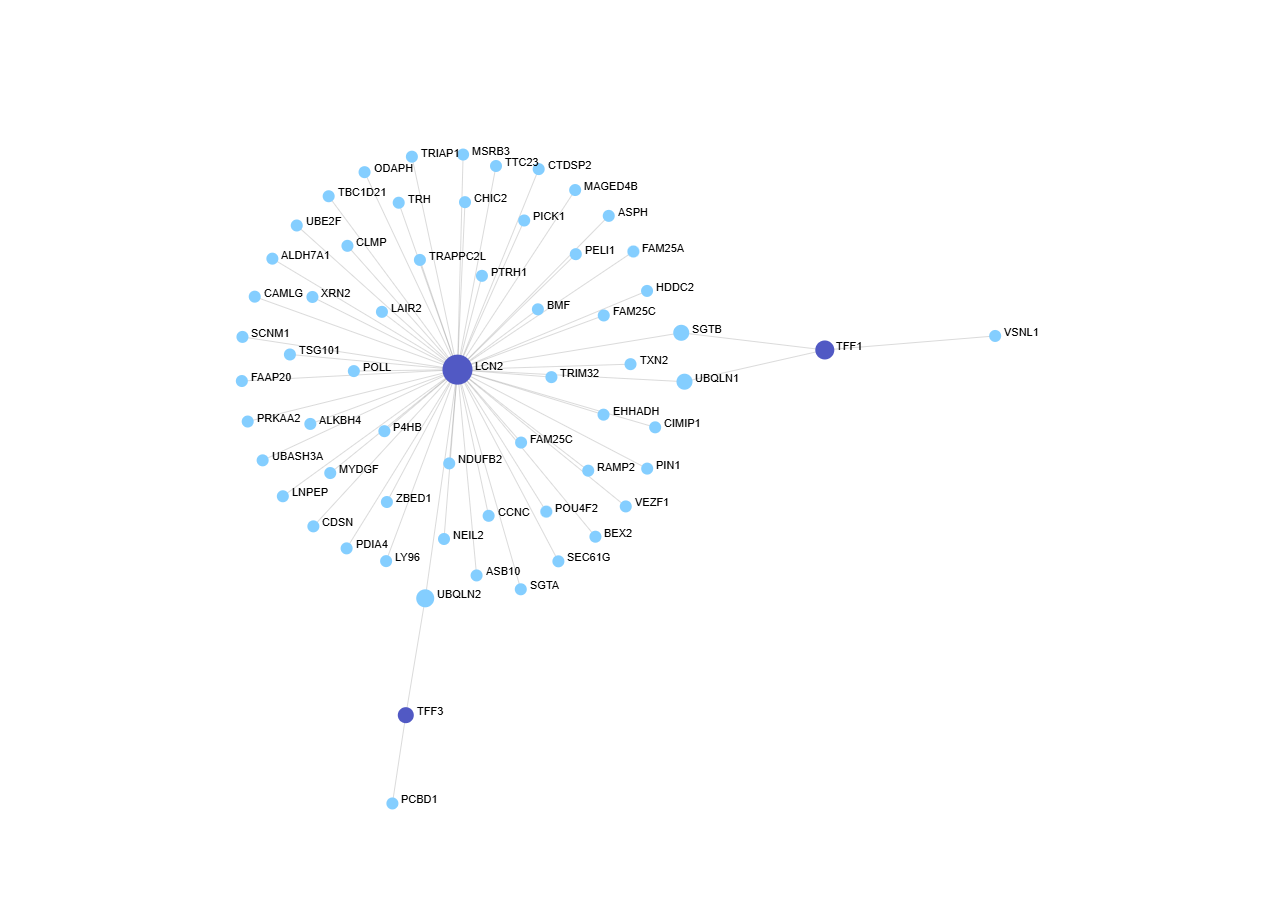

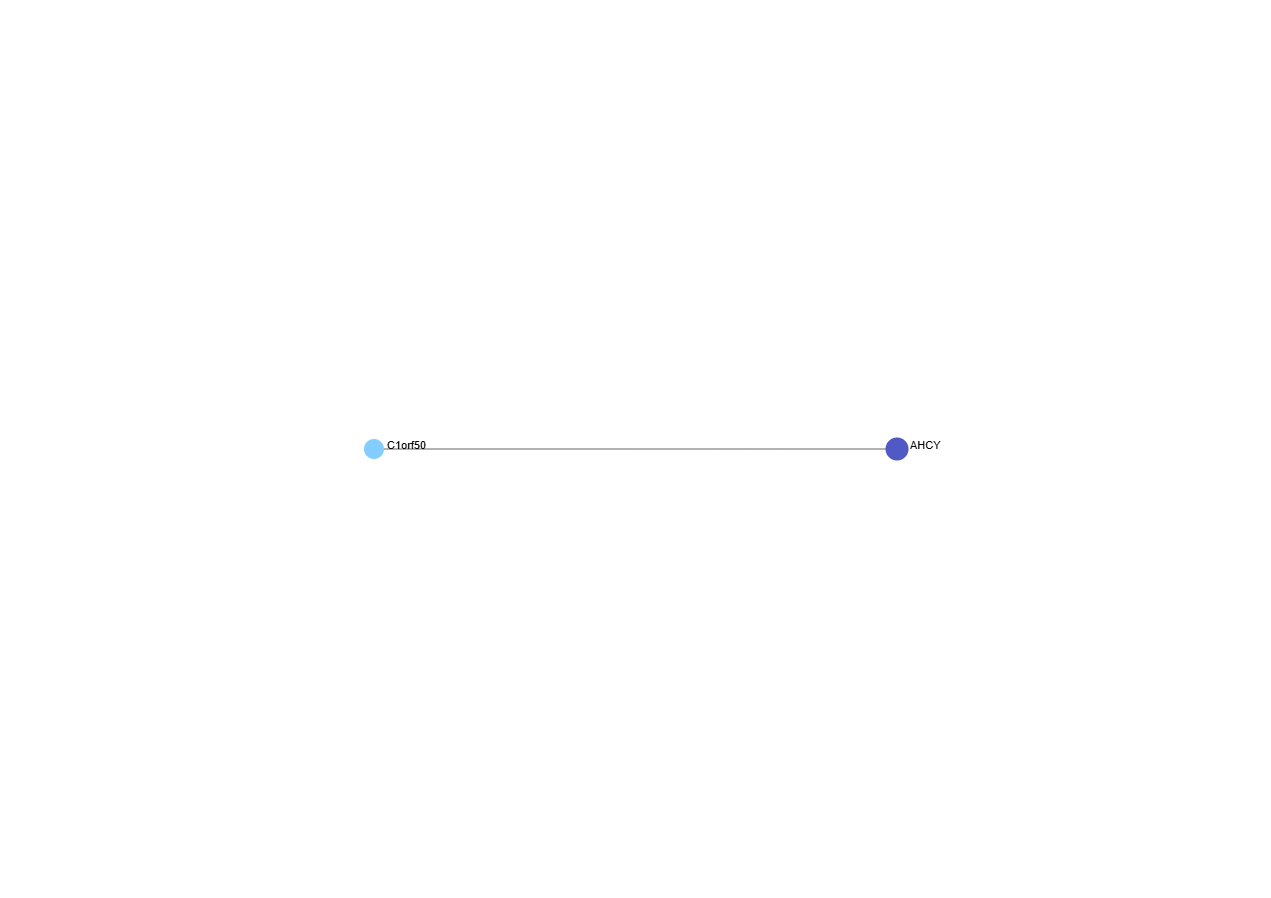

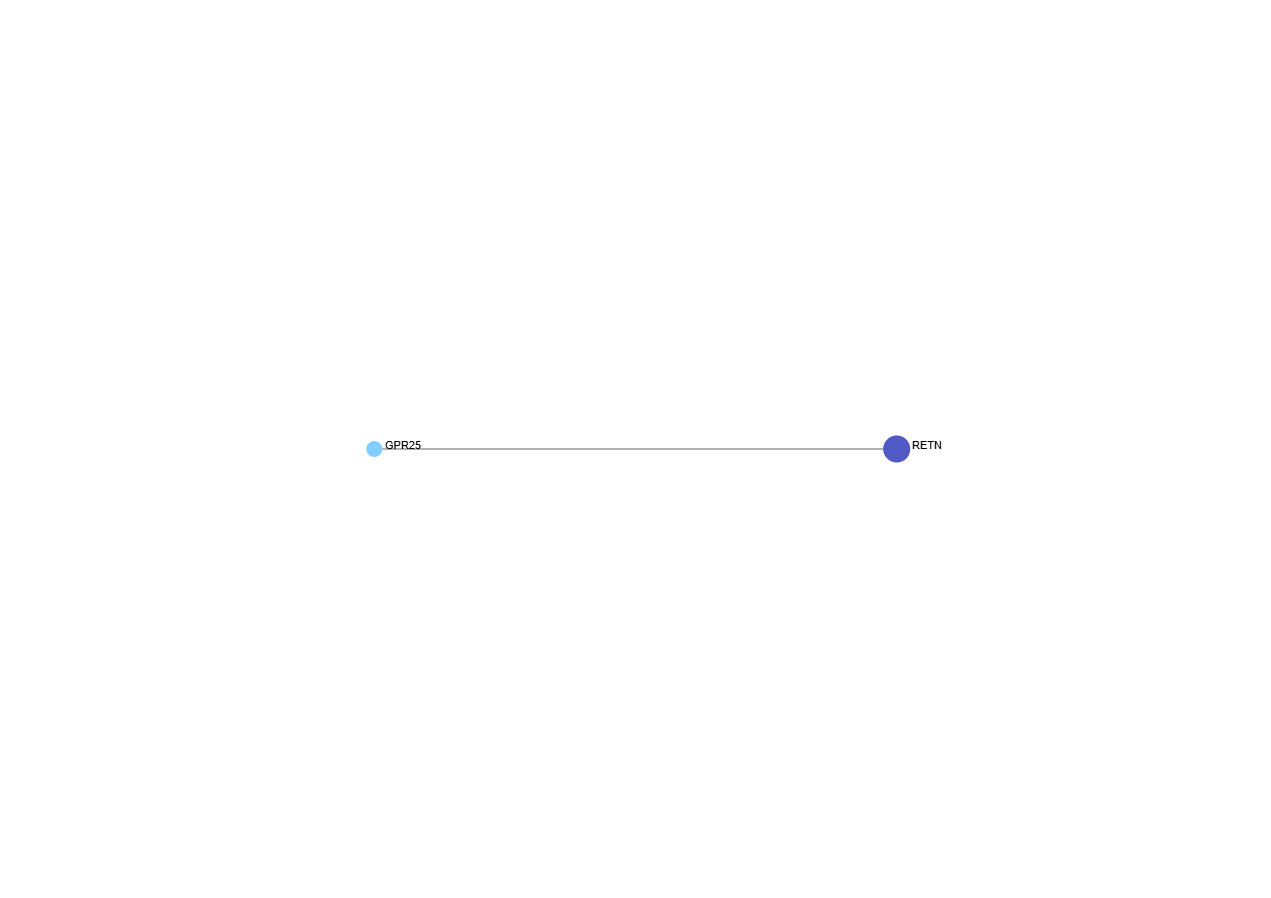


**A**

**B**

**C**

***Supplementary Figure 2: HuRI PPI Network.***

*PPI network generated using HuRI for seven CRC associated seed proteins (TFF3, TFF1, AHCY, RETN, LCN2, SELE, CEACAM5). The network consisted of 66 nodes and 65 edges across three subnetworks with five out of the seven proteins. (A) The largest subnetwork contained 62 nodes and 62 edges, incorporating LCN2, TFF1 and TFF3. LCN2 exhibited the highest centrality within this subnetwork, whereas TFF1 and TFF3 were peripherally located. Non seed proteins SGTB, UBQLN1 and UBQLN2 were placed between seed nodes. (B) RETN and (C) AHCY formed small subnetworks with a single direct interaction. SELE and CEACAM5 were not detected.*

*Dark blue nodes indicate seed proteins, light blue nodes represent proteins. Grey edges represent direct protein-protein interactions. Network visualised using OmicsNet.*


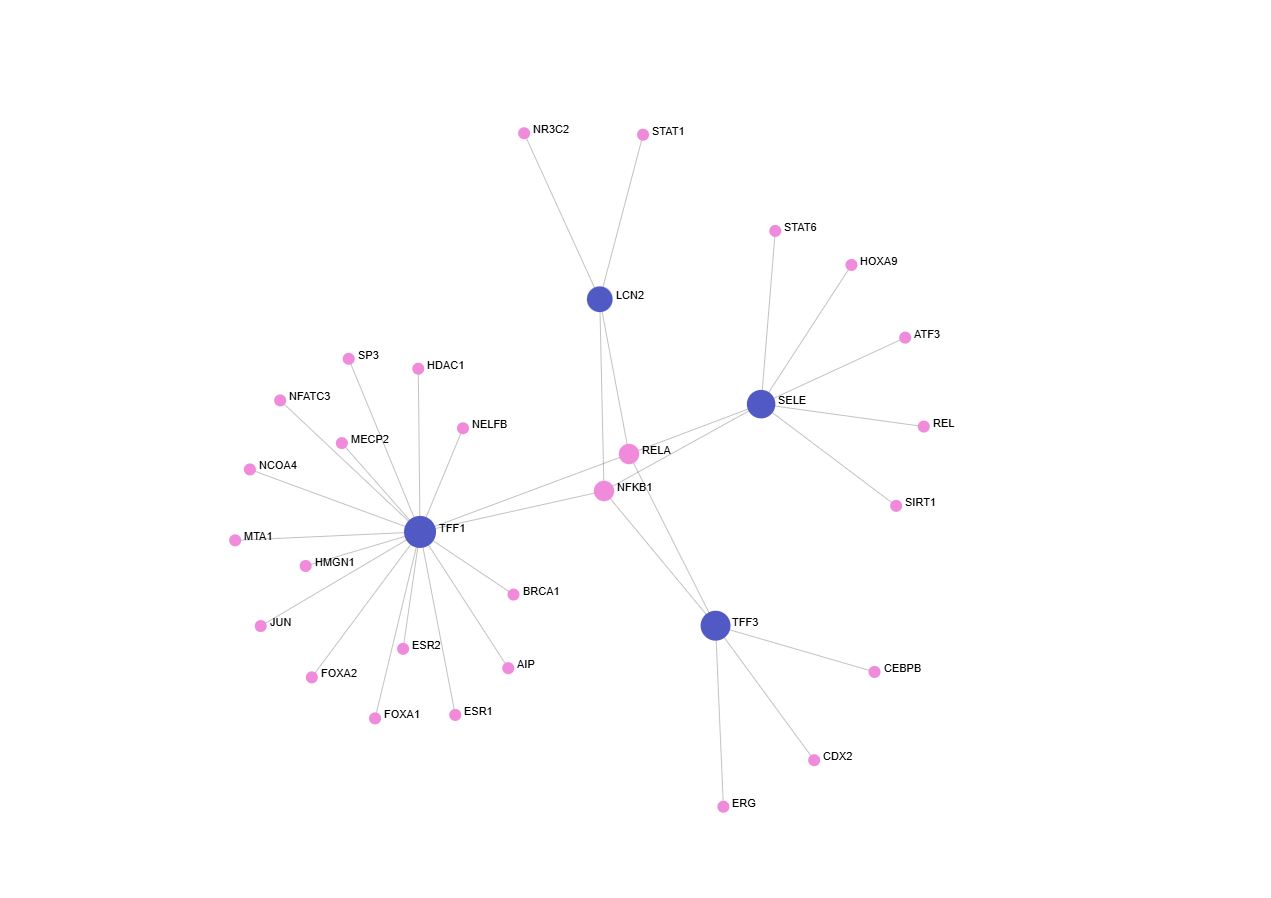

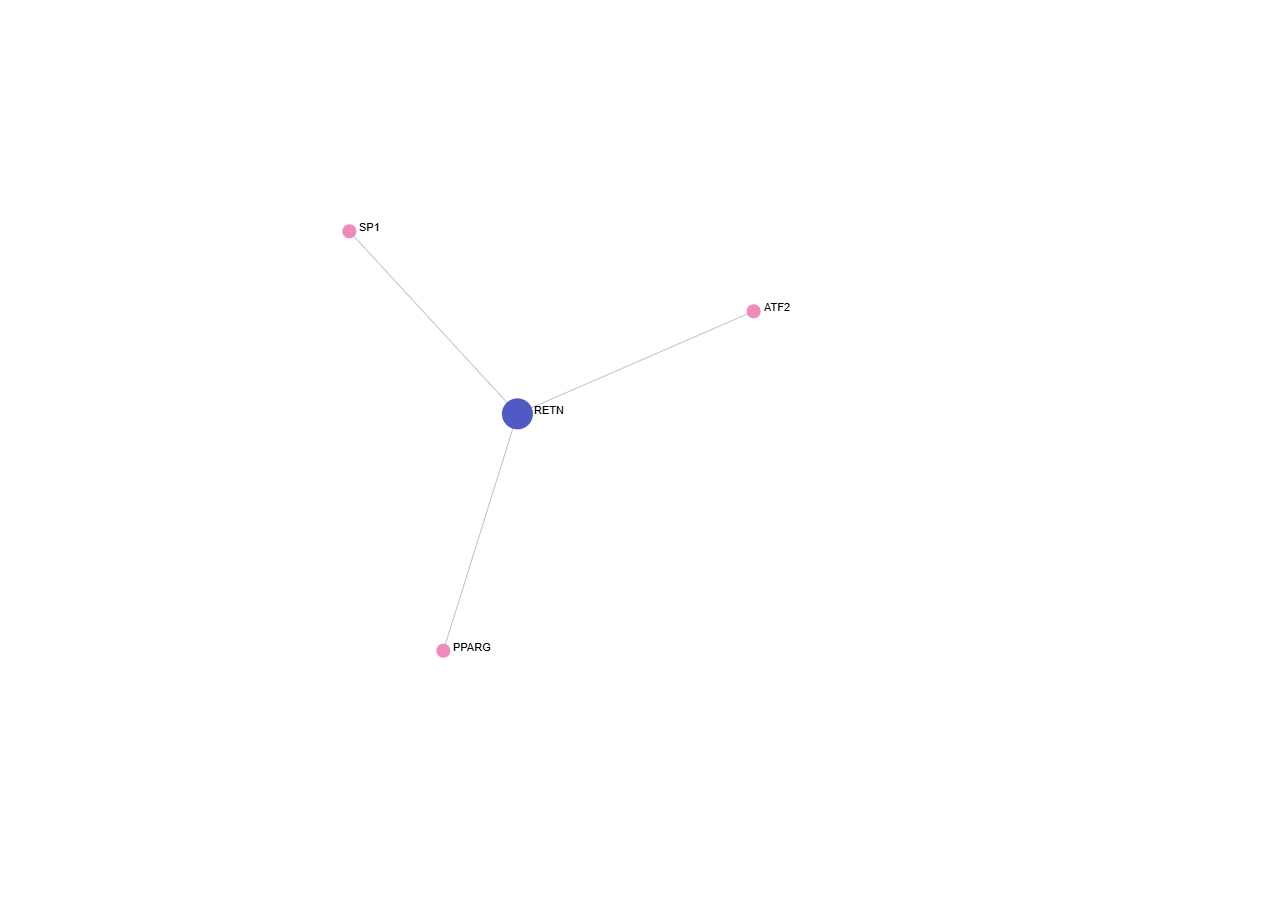

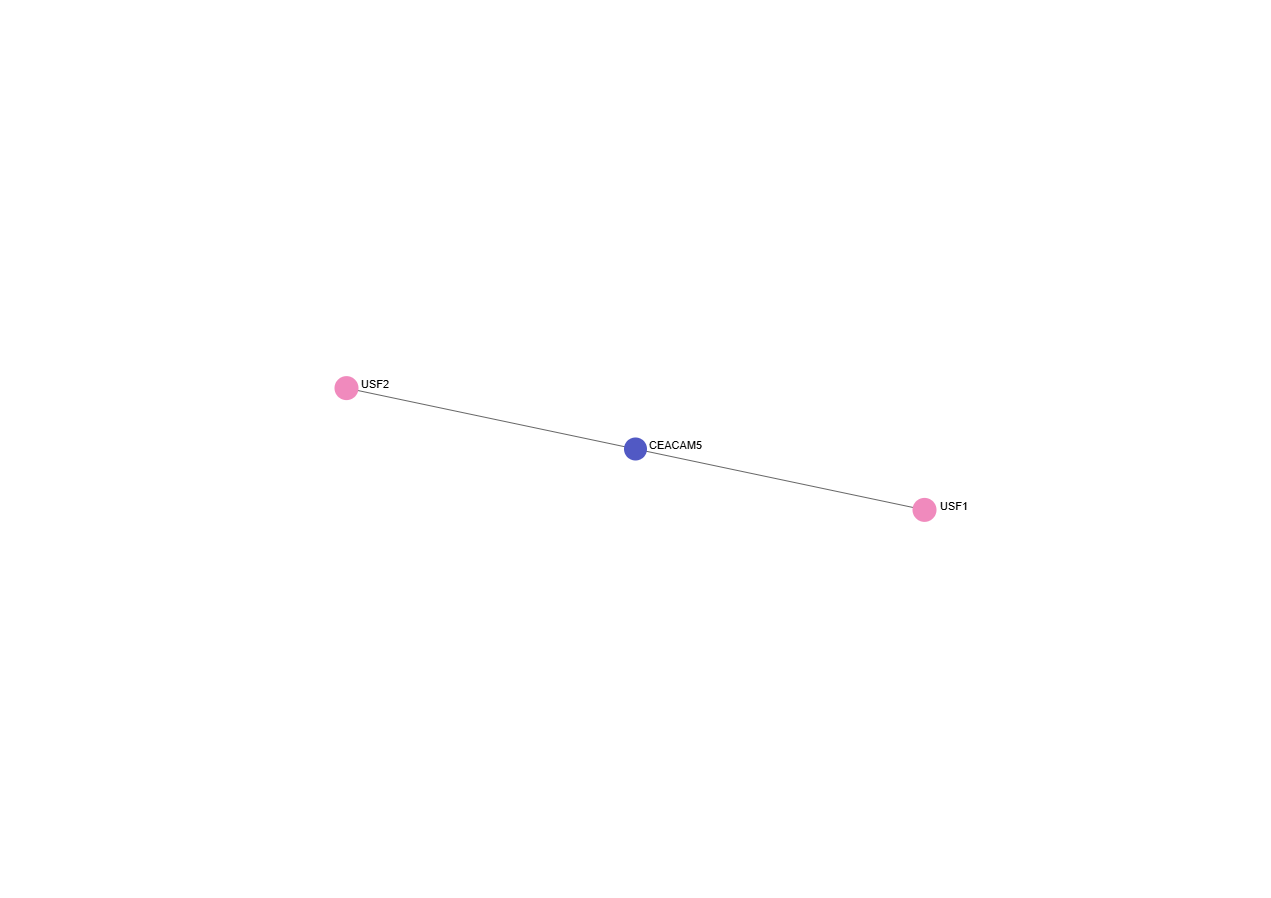


**A**

**B**

**C**

***Supplementary Figure 3: TTRUST transcription factor-protein interaction network.***

*Transcription factor-protein interaction network generated using the TTRUST database for seven CRC associated seed proteins (TFF3, TFF1, AHCY, RETN, LCN2, SELE, and CEACAM5). The output network consisted of 38 nodes and 38 edges across three subnetworks with six of the proteins, AHCY was not involved. (A) The largest subnetwork included TFF1, SELE, LCN2 and TFF3, each connected to overlapping transcription factors RELA and NF-κB. TFF1 emerged as the most central seed node in this cluster. (B) RETN formed a smaller subnetwork with three direct transcription factors interactions, ATF2, SP1 and PPARG. (C) CRACAM5 was connected to USF1 and USF2 in an isolated subnetwork. Dark blue nodes represent seed proteins and pink nodes represent transcription factors. Network visualised using OmicsNet.*


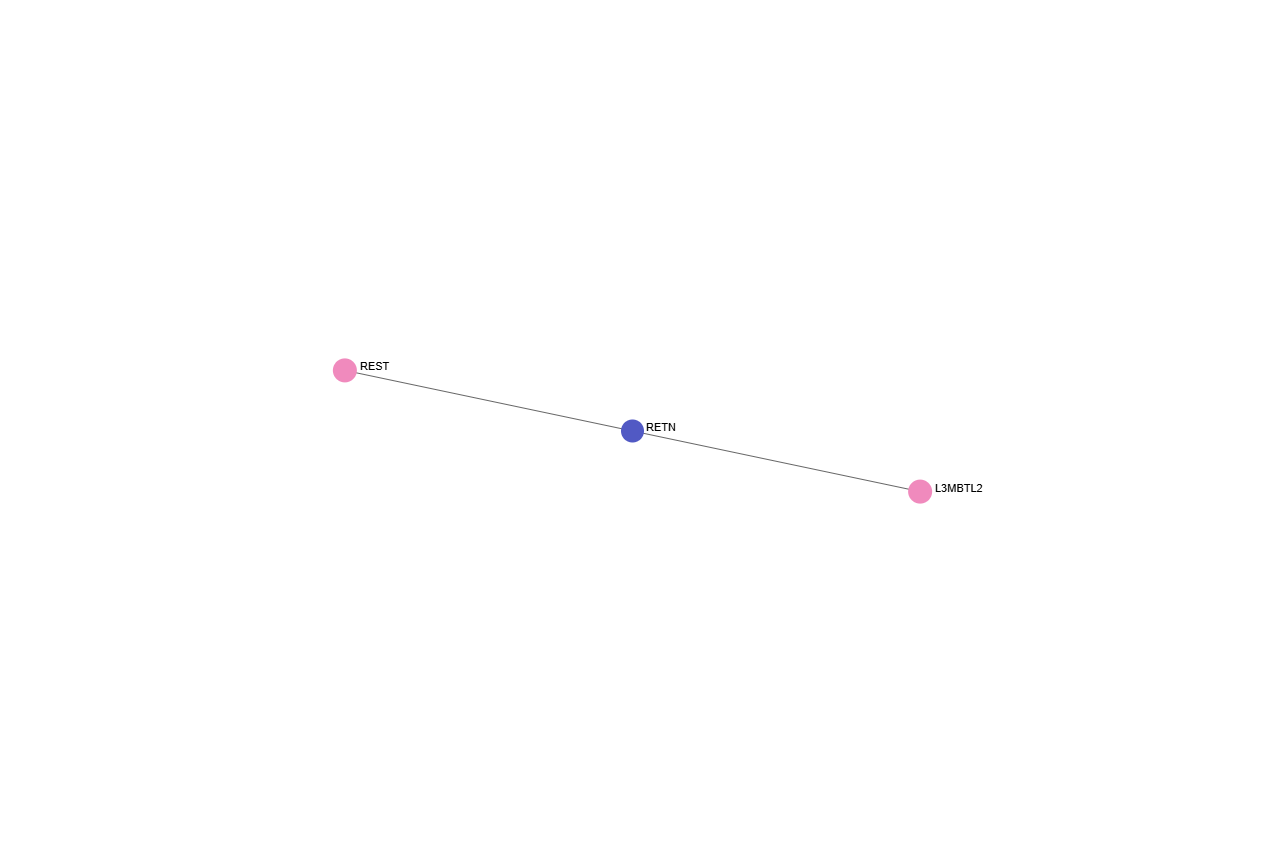

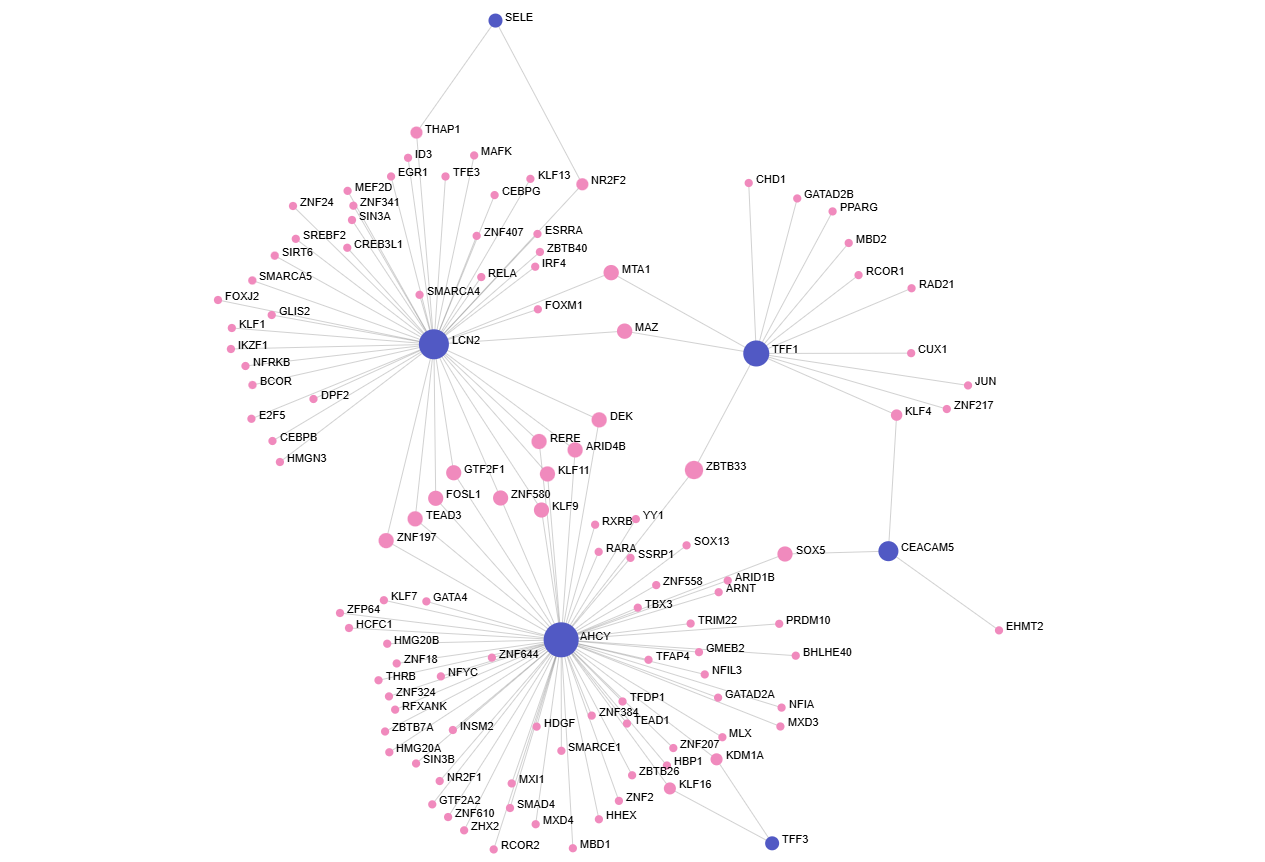


***Supplementary Figure 4: ENCODE Transcription Factor-Target Interaction Network***

*Transcription factor-target interaction network generated using the ENCODE database for seven CRC-associated seed proteins (TFF3, TFF1, AHCY, RETN, LCN2, SELE, CEACAM5). The complete network consisted of 122 nodes and 134 edges distributed across two subnetworks. (A) The main subnetwork included six seed proteins (AHCY, LCN2, TFF1, TFF3, SELE and CEACAM5) connected to 113 transcription factors through 132 interactions. AHCY was the most connected node in the network. (B) RETN formed an isolated subnetwork, displaying only two TF interactions (REST and LSMBTL2), indicating minimal transcriptional integration. Dark blue nodes represent seed proteins and pink nodes represent transcription factors. Network visualised using OmicsNet.*


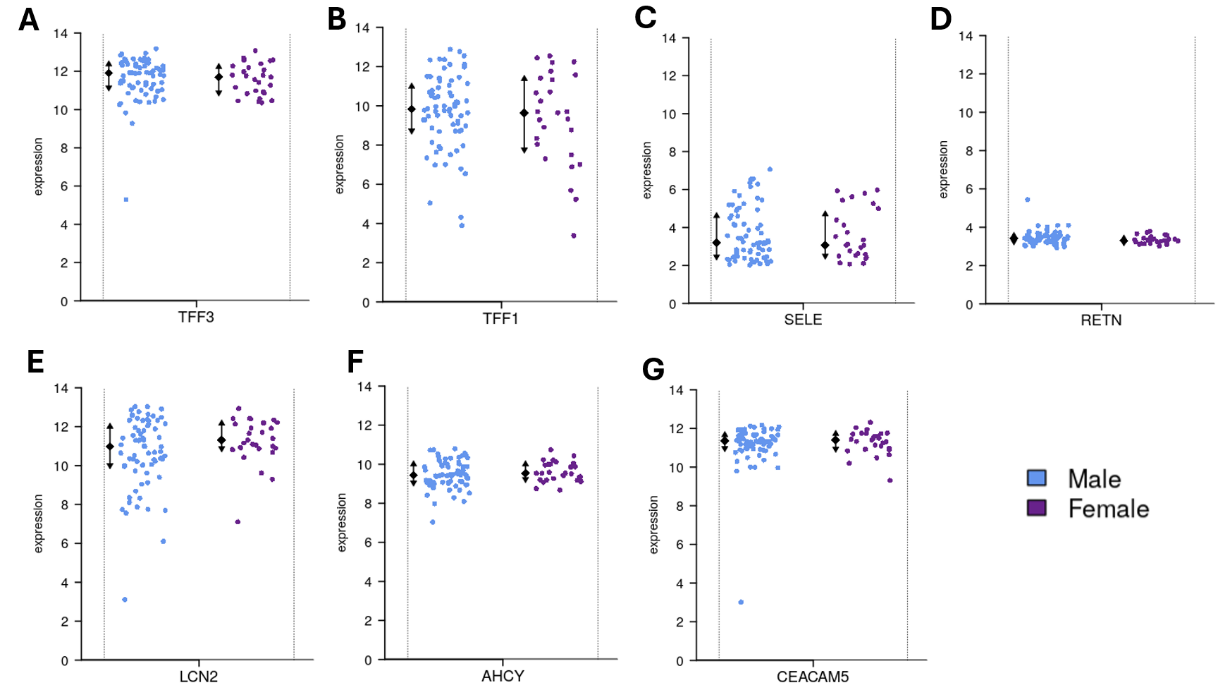


***Supplementary Figure 5: Gender Based Differences in Gene Expression of CRC Associated Proteins in Stage II MSS Colon Cancer.***

*Transcriptomic expression levels of seven colorectal cancer-associated genes, (A) TFF3, (B) TFF1, (C) SELE, (D) RETN, (E) AHCY, (F) LCN2 and (G) CEACAM, were analysed in male (blue, n = 71) and female (purple, n = 21) tumour samples using the Colonomics dataset. No statistically significant sex-based differences were observed for any of the genes (p > 0.05). Each point represents an individual sample. Black error bars represent the mean ± (SD) for each group. Visualisation was performed using the Colonomics platform*


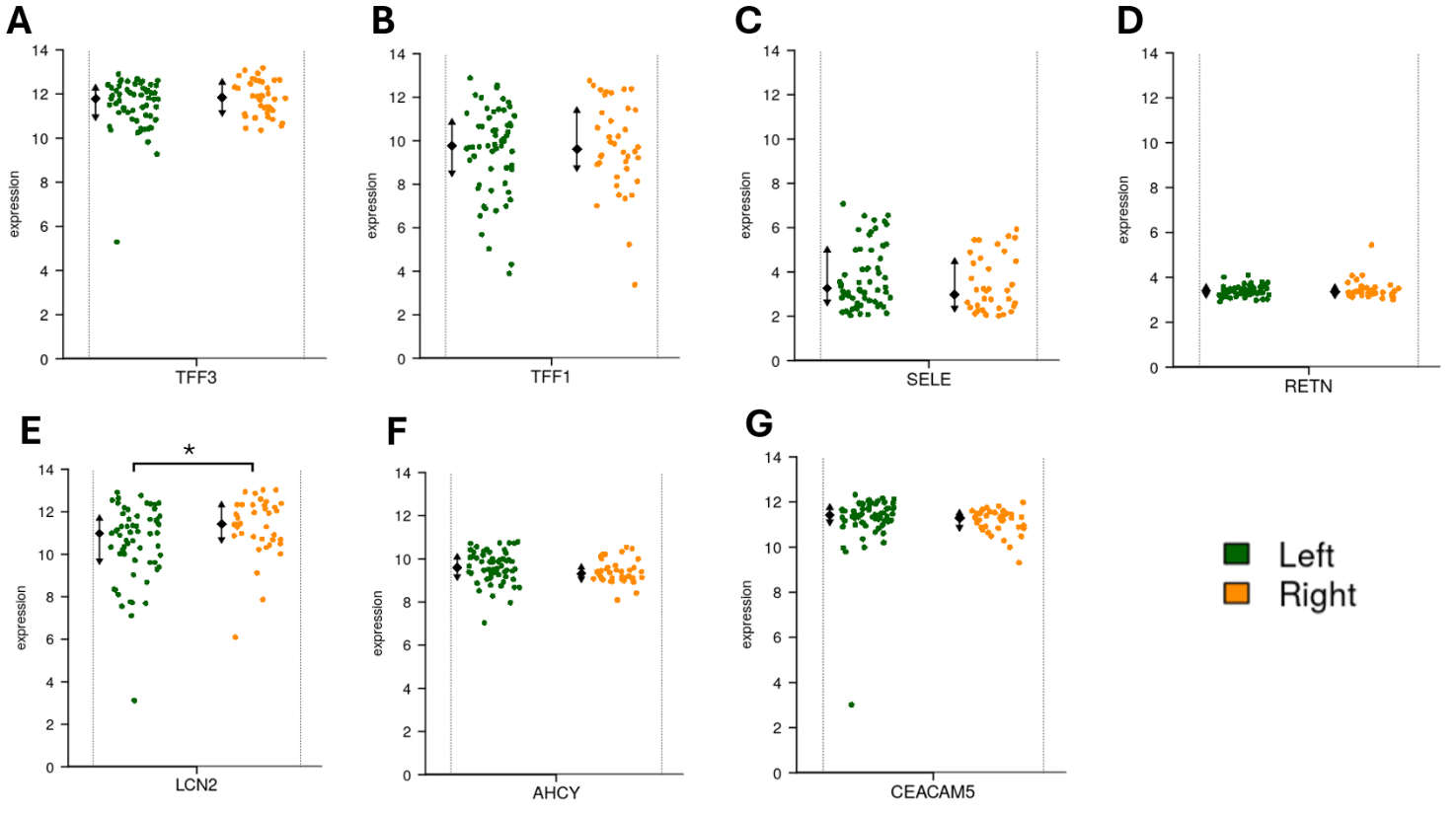


***Supplementary Figure 6: Gene expression of CRC associated proteins in left and right sided stage II MSS colon tumours.***

*Transcriptomic expression of seven CRC associated genes (A) TFF3, (B) TFF1, (C) SELE, (D) RETN, (E) AHCY, (F) LCN2 and (G) CEACAM5 was assessed based on tumour location in patients with stage II MSS colon cancer. Only LCN2 displayed a significant increase in expression between left and right sided tumours. Each dot represents a patient sample; the left side is represented by green dots (n=60) and the right side is represented by orange dots (n=38). Black error bars represent the mean ± (SD) for each group. Visualisation was performed using the Colonomics platform. * p<0.05.*


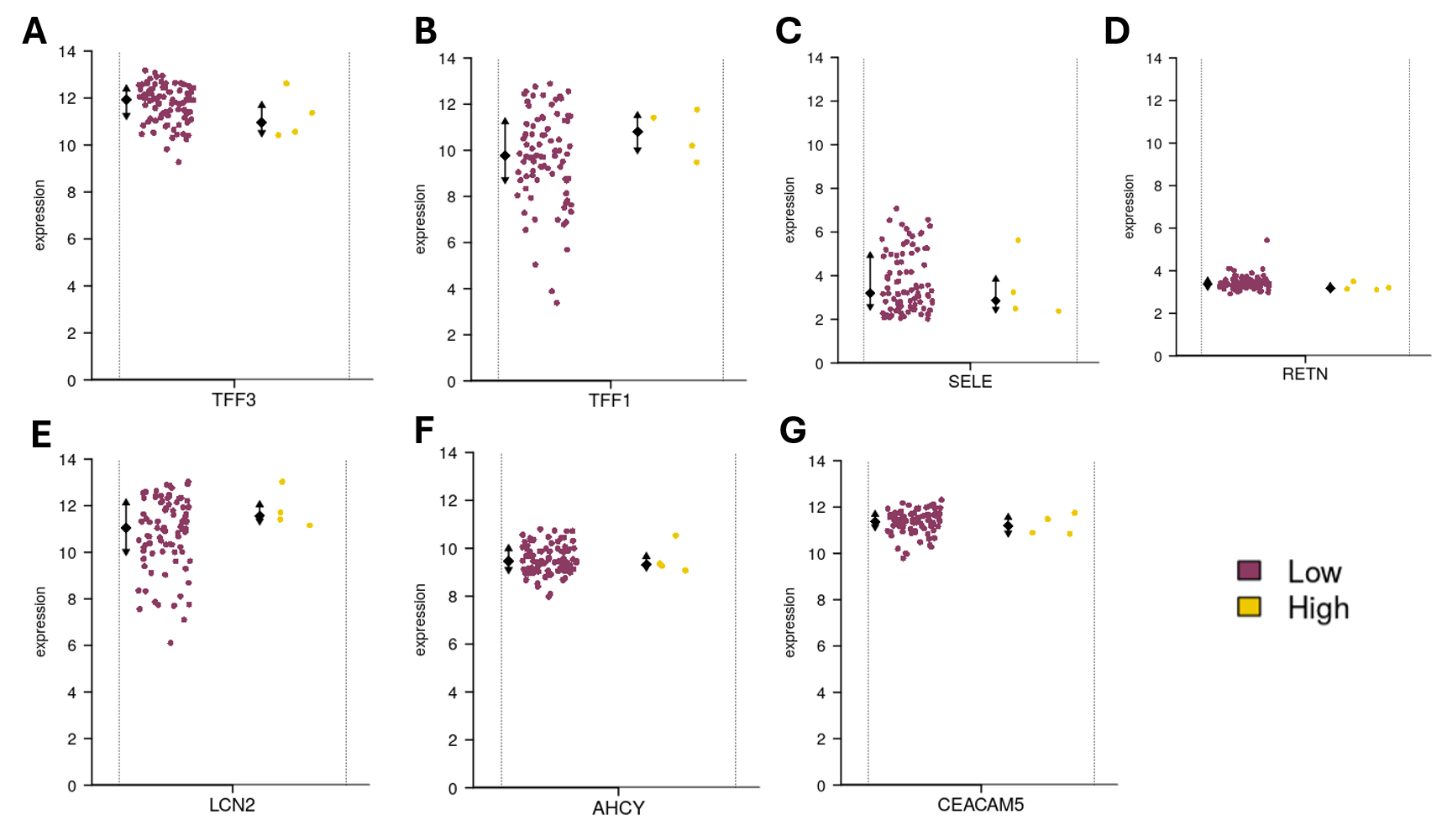


***Supplementary Figure 7****: Gene expression of seven CRC associated proteins* by CIMP status in stage II MSS colon cancer. Transcriptomic expression levels of seven CRC associated genes *(A) TFF3, (B) TFF1, (C) SELE, (D) RETN, (E) AHCY, (F) LCN2 and (G) CEACAM5 were compared between tumours classified as CIMP-low (purple, n = 86) and CIMP-high (yellow, n = 4). No statistically significant differences between CIMP status were found. Black error bars represent the mean ± (SD) for each group. Visualisation was performed using the Colonomics platform.*


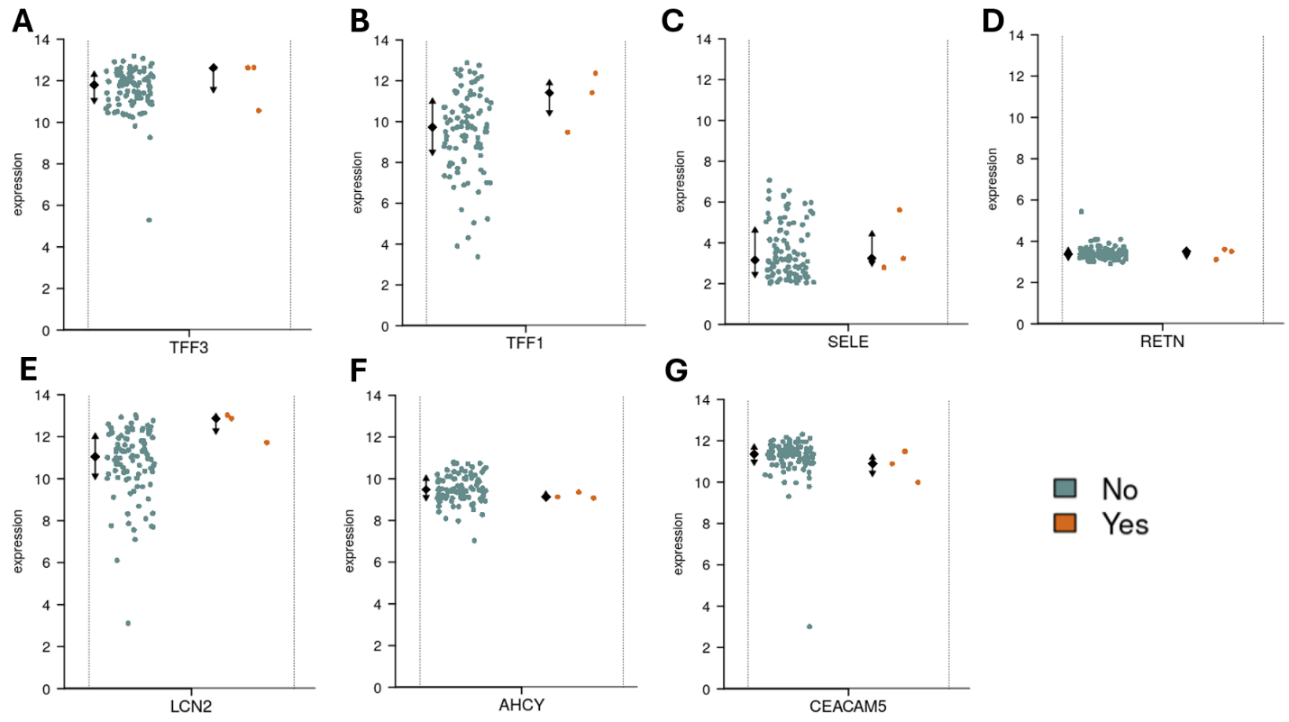


***Supplementary Figure* 8**: *Gene expression of seven CRC associated proteins grouped by BRAF V600 mutation status in stage II MSS colon cancer. Transcriptomic expression of seven colorectal cancer-associated genes, (A) TFF3, (B) TFF1, (C) SELE, (D) RETN, (E) AHCY, (F) LCN2 and (G) CEACAM5, were analysed according to BRAF V600 mutation status in stage II microsatellite stable (MSS) colon cancer samples. Grey-green dots represent patients without the mutation (n = 95), while orange dots represent patients with a BRAF V600 mutation (n = 3). . No statistically significant results were observed. Black error bars represent the mean ± (SD) for each group. Visualisation was performed using the Colonomics platform.*

**
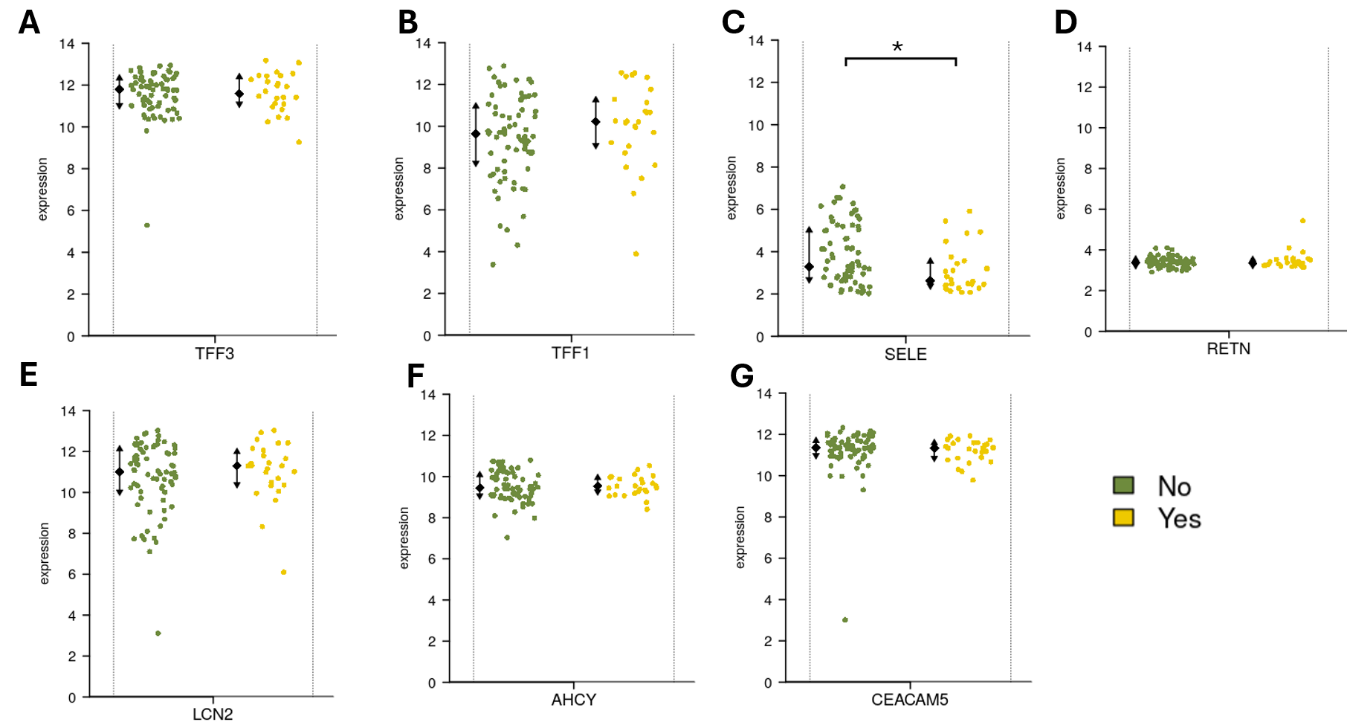
**

***Supplementary Figure 9*** *Gene expression of seven CRC-associated proteins stratified by KRAS mutation status in stage II MSS colon cancer.
Transcriptomic expression levels of seven CRC associated genes TFF3, TFF1, SELE, RETN, LCN2, AHCY, and CEACAM5 were compared based on the presence or absence of KRAS mutations in stage II MSS colon cancer tumour samples. Green dots represent samples without KRAS mutations (n = 72), while yellow dots represent samples with KRAS mutations (n = 26). Only SELE demonstrated statistically significant differences between KRAS mutations. Each dot corresponds to an individual patient sample. Black error bars represent the mean ± SD for each group. Visualisation was performed using the Colonomics platform. * p<0.05.*

*
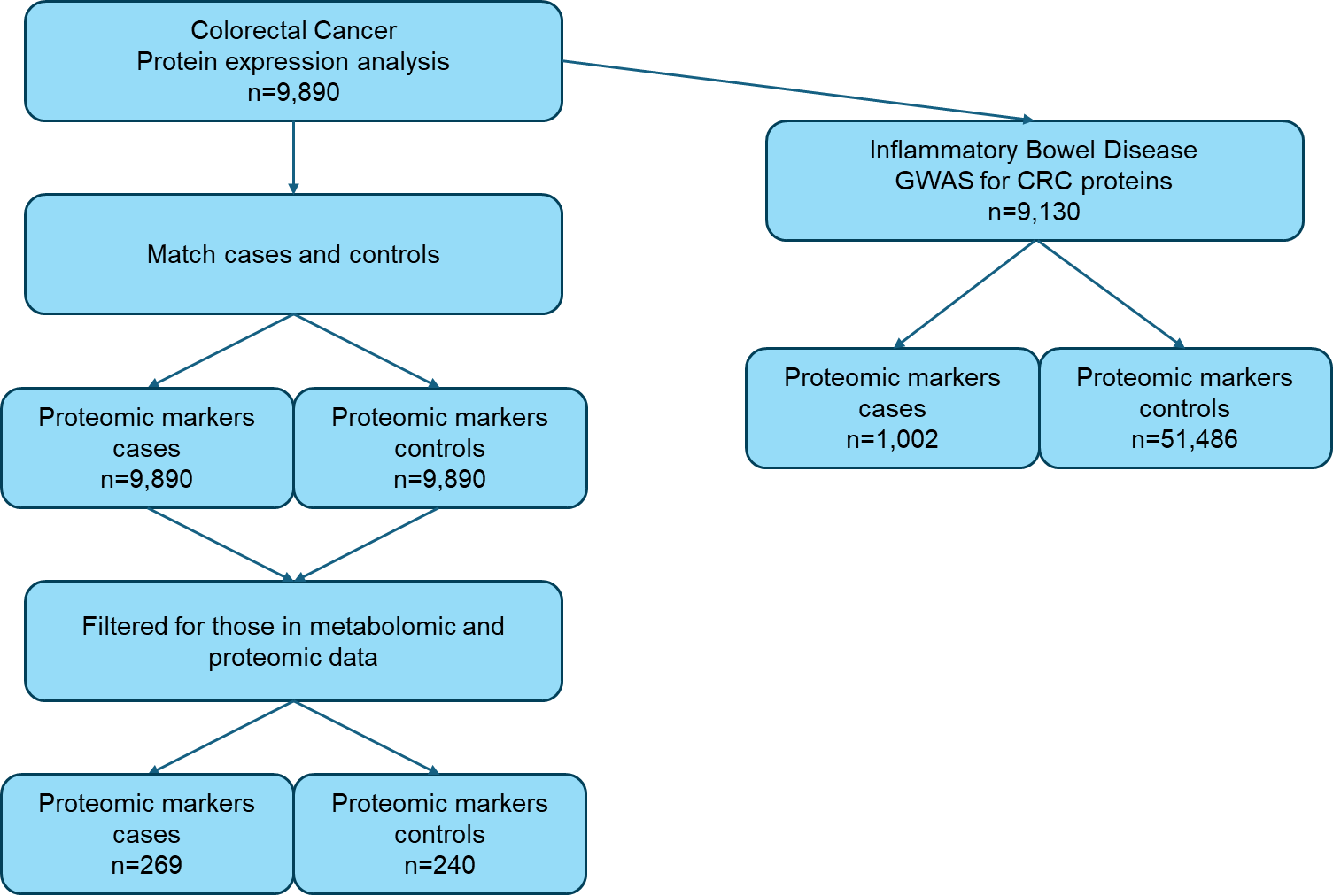
*

***Supplementary Figure 10 :*** *Workflow showing selection of cases and controls in two separate related analyses. On the left is protein expression analysis in colorectal cancer. This followed the curation steps followed in Kollampallath (2025). On the right are details for GWAS analysis for the seven proteins in the CRC analysis. These were tested for cases and controls of inflammatory bowel disease and their results compared statistically.*
